# Biological Activity of Triazolopyrimidine Copper(II) Complexes Modulated by an Auxiliary N-N-Chelating Heterocycle Ligands

**DOI:** 10.3390/molecules26226772

**Published:** 2021-11-09

**Authors:** Lavinia L. Ruta, Ileana C. Farcasanu, Mihaela Bacalum, Mina Răileanu, Arpad Mihai Rostas, Constantin Daniliuc, Mariana Carmen Chifiriuc, Luminița Măruțescu, Marcela Popa, Mihaela Badea, Emilia Elena Iorgulescu, Rodica Olar

**Affiliations:** 1 Department of Organic Chemistry, Biochemistry and Catalysis, Faculty of Chemistry, University of Bucharest, 90–92 Panduri Str., 050663 Bucharest, Romania; lavinia.ruta@chimie.unibuc.ro; 2 Department of Life and Environmental Physics, Horia Hulubei National Institute for Physics and Nuclear Engineering, 30 Reactorului Str., 077125 Măgurele, Romania; bmihaela@nipne.ro (M.B.); mina.raileanu@nipne.ro (M.R.); 3 Department of Electricity, Solid State and Biophysics, Faculty of Physics, University of Bucharest, 405A Atomiştilor Str., 077125 Măgurele, Romania; 4 Laboratory of Atomic Structures and Defects in Advanced Materials, National Institute of Materials Physics, 405A Atomiştilor Str., 077125 Măgurele, Romania; arpad.rostas@infim.ro; 5 Organisch-Chemisches Institute, Westfälische Wilhelms-Universität Münster, Corrensstrasse 40, 48149 Münster, Germany; constantin.daniliuc@uni-muenster.de; 6 Department of Microbiology, Faculty of Biology, University of Bucharest, 1–3 Aleea Portocalelor Str., 060101 Bucharest, Romania; carmen.chifiriuc@bio.unibuc.ro (M.C.C.); luminita.marutescu@bio.unibuc.ro (L.M.); marcela.popa@bio.unibuc.ro (M.P.); 7 Department of Inorganic Chemistry, Biochemistry and Catalysis, Faculty of Chemistry, University of Bucharest, 90–92 Panduri Str., 050663 Bucharest, Romania; mihaela.badea@chimie.unibuc.ro; 8 Department of Analytical Chemistry, Faculty of Chemistry, University of Bucharest, 90–92 Panduri Str., 050663 Bucharest, Romania; emilia-elena.iorgulescu@chimie.unibuc.ro

**Keywords:** copper(II) complex, 1,2,4-triazolo[1,5-*a*]pyrimidine, cytotoxicity, biofilm, metallonuclease activity, DNA intercalation

## Abstract

Novel complexes of type [Cu(N-N)(dmtp)_2_(OH_2_)](ClO_4_)_2_·dmtp ((**1**) N-N: 2,2′-bipyridine; (**2**) L: 1,10-phenantroline and dmtp: 5,7-dimethyl-1,2,4-triazolo[1,5-*a*]pyrimidine) were designed in order to obtain biologically active compounds. Complexes were characterized as mononuclear species that crystallized in the space group P-1 of the triclinic system with a square pyramidal geometry around the copper (II). In addition to the antiproliferative effect on murine melanoma B16 cells, complex (**1**) exhibited low toxicity on normal BJ cells and did not affect membrane integrity. Complex (**2**) proved to be a more potent antimicrobial in comparison with (**1**), but both compounds were more active in comparison with dmtp—both against planktonic cells and biofilms. A stronger antimicrobial and antibiofilm effect was noticed against the Gram-positive strains, including methicillin-resistant *Staphylococcus aureus* (MRSA). Both electron paramagnetic resonance (EPR) and *Saccharomyces cerevisiae* studies indicated that the complexes were scavengers rather than reactive oxygen species promoters. Their DNA intercalating capacity was evidenced by modifications in both absorption and fluorescence spectra. Furthermore, both complexes exhibited nuclease-like activity, which increased in the presence of hydrogen peroxide.

## 1. Introduction

The triazolopyrimidine derivatives are valuable pharmacophores based on their resemblance to purine bases. As result, several compounds bearing diverse substituents were developed as species with relevant biological activity [1]. The most recent findings in the field describe triazolopyrimidines as agents with potent antiproliferative activity by inducing G0/G1 phase arrest [2] or cellular apoptosis [3], as species with anti-HIV activity [4], as SARS-CoV-2 main protease inhibitors [5], and as antimicrobials [6,7]. Additionally, it is believed that triazolopyrimidines are promising candidates for treating Alzheimer’s disease and related neurodegenerative tauopathies [8].

A promising activity, comparable with that of standard anticancer drugs, against a panel of cancer cell lines was reported for [1,2,4]triazolo[1,5-*a*]pyrimidine derivatives (tpds). Among these, some pyrimidine ring-disubstituted species exhibit a good activity against colon (HCT116) [9], human promyelocytic leukemia (HL60) [10], gastric (MGC-803), esophageal (EC 109), lung (A549 and PC-9) [11], liver (HepG2) [12] and breast (MCF-7) [12,13] cancer cell lines. It is worth mentioning that most of these lines are not sensitive to inorganic drugs such as cisplatin and related derivatives.

A significant antibacterial activity, comparable with that of standard antimicrobials against *Staphylococcus aureus*, *Escherichia coli* [14,15], *Bacillus subtilis* [14] and *Pseudomonas aeruginosa* [15] strains was also reported for some substituted tpds.

Considering its biological potency, the tp scaffold is often used to develop biologically active complexes, with a net preference for Cu(II) against other metal ions. Copper(II) exhibits particular traits, such as borderline acid characteristics or stereochemical and oxidation state versatility [16]. Moreover, most of the Cu(II) complexes with tps were reported as having antiparasitic [17], antitumor, or antimicrobial activity [18].

Among the antiparasitic compounds, [Cu_2_(μ-7atp)_4_Cl_2_]Cl_2_·4H_2_O, [Cu_2_(μ-7atp)_4_(H_2_O)_2_](NO_3_)·4H_2_O (7atp: 7-amino-1,2,4-triazolo[1,5-*a*]pyrimidine) [19], [Cu_2_(dmtp)_4_Cl_4_]2H_2_O [20], [Cu(dmtp)_4_(H_2_O)_2_](ClO_4_)_2_·2H_2_O [21], and [Cu(dmtp)_2_(NO_3_)_2_(H_2_O)] (dmtp: 5,7-dimethyl-1,2,4-triazolo[1,5-*a*]pyrimidine) [22] showed a substantial in vitro activity against *Leishmania* spp. and *Trypanosoma cruzi*. Additionally, low toxicity against macrophage host cells was observed for all complexes.

A good antiproliferative activity against *L. infantum* and *L. braziliensis* was also observed for [Cu(HmtpO)_2_(H_2_O)_3_](ClO_4_)_2_H_2_O and {[Cu(HmtpO)_2_(H_2_O)_2_](ClO_4_)_2_2HmtpO}_n_ (HmtpO: 5-methyl-1,2,4-triazolo[1,5-*a*]pyrimidin-7(4H)-one)—complexes that affect the metabolism of the parasites at the level of both NAD+/NADH balance and organelle membranes, causing the cell death [23].

Moreover, complexes with mixed ligands such as [Cu(H_2_O)(phen)(tp)_2_](ClO_4_)_2_·H_2_O and [Cu(NO_3_)(H_2_O)(phen)(tp)](NO_3_) (phen: 1,10-phenanthroline) [24] inhibit in vitro epimastigote forms of *T. cruzi* and promastigotes of *L. peruviana,* while [Cu(dmtp)_2_(bpym)(H_2_O)_2_](ClO_4_)_2_2H_2_O and [Cu_2_(tp)_2_(bpym)_2_(μ-bpym)(ClO_4_)_2_](ClO_4_)_2_ (bpym: 2,2′-bipyrimidine) exhibit activity against the same parasites, which cause leishmaniasis and Chagas disease, respectively [25].

A broad spectrum of antimicrobial activity was demonstrated by [Cu_2_(pmtp)_2_Cl_4_(OH_2_)_2_] [26], [Cu(pmtp)(CH_3_COO)_2_]0.5H_2_O [27] and [Cu(pmtp)(OH_2_)_3_](ClO_4_)3H_2_O (pmtp: 5-phenyl-7-methyl-1,2,4-triazolo[1,5-*a*]pyrimidine) [28] against both planktonic and biofilm-embedded strains. It is worth mentioning the activity of all complexes against methicillin-resistant *S. aureus* (MRSA). Moreover, complexes with mixed ligands [Cu(N-N)_2_(pmtp)](ClO_4_)_2_ (N-N: 2,2′-bipyridine (bpy) and phen) showed antibacterial potential against several bacterial strains, including MRSA, the extended-spectrum beta-lactamase (ESBL)-producing *E. coli* 5, and the multi-drug-resistant *P. aeruginosa* 9027, both in planktonic and biofilm growth state [18].

Among these species, [Cu(pmtp)(CH_3_COO)_2_]0.5H_2_O and [Cu(pmtp)(OH_2_)_3_](ClO_4_)3H_2_O also induce a decrease in the DNA content of cells in the G0/G1 phase in a human colon adenocarcinoma cell line (HT 29) [27], while [Cu(N-N)_2_(pmtp)](ClO_4_)_2_ exhibited excellent activity against B16 murine melanoma cells, that in addition, is accompanied by a lack of cytotoxicity against healthy BJ cells [18].

Concerning species with N-N derivatives, we have recently shown that {[Cu(bpy)_2_(μ_2_OClO_3_)]ClO_4_}_n_ exhibits a selective cytotoxicity against B16 cells while [Cu(phen)_2_(OH_2_)](ClO_4_)_2_ proved to be a very potent antibacterial agent against both susceptible and resistant Gram-positive and Gram-negative strains, in planktonic or biofilm growth states [29]. By combining these N-N heterocycles with a disubstituted tp derivative we succeded in improving the antimicrobial activity of Cu(II) complexes [18].

In an attempt to modulate the biological activity of dmtp Cu(II) complexes through such N-N derivatives, we present here new complexes with mixed ligands of [Cu(N-N)(dmtp)_2_(OH_2_)](ClO_4_)_2_dmtp (N-N: bpy (**1**) and phen (**2**)) type that were fully characterized by single-crystal X-ray analysis and spectroscopic methods. In addition, data regarding the antitumor and antimicrobial activity of these complexes are also included, together with that concerning their potential mechanisms of action.

## 2. Results and Discussion

**Scheme 1:**
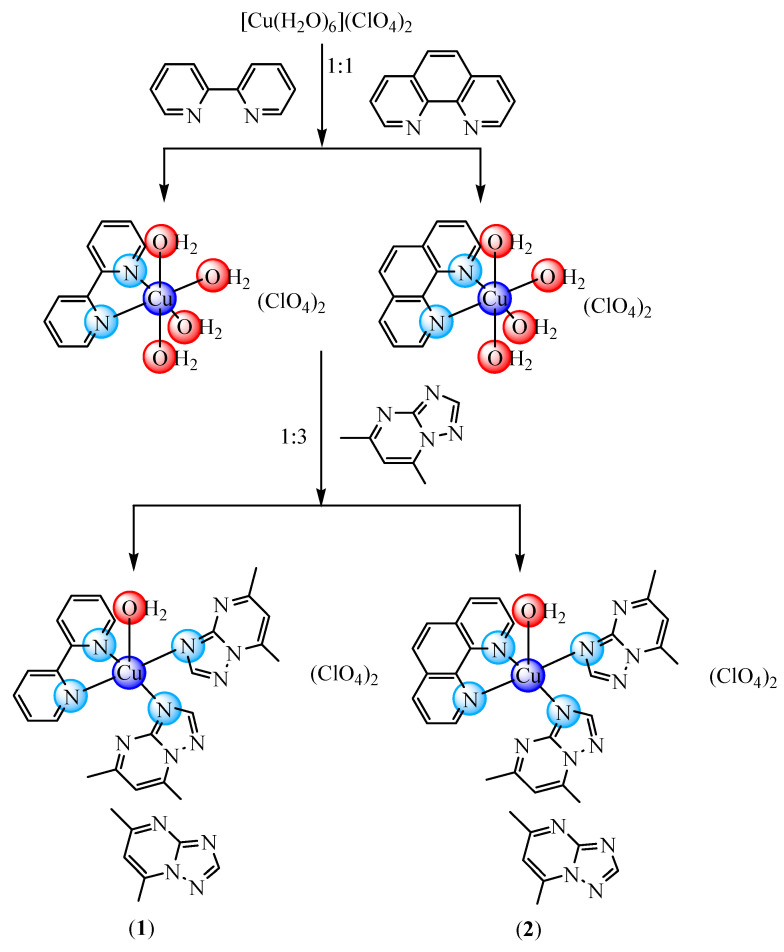
The route of complexes [Cu(bpy)(dmtp)_2_(OH_2_)](ClO_4_)_2_·dmtp (**1**) and [Cu(phen)(dmtp)_2_(OH_2_)](ClO_4_)_2_·dmtp (**2**) synthesis.

A new series of copper(II) complexes with mixed ligands 2,2′-bipyridine (bpy) or 1,10-phenanthroline (phen) and 5,7-dimethyl-1,2,4-triazolo[1,5-*a*]pyrimidine (dmtp) were synthesized by stepwise reactions, as depicted in Scheme 1.

First, the [Cu(N-N)]^2+^ intermediates were prepared, and then the dmtp was added in a suitable molar ratio. The compounds were characterized as mononuclear species by single-crystal X-ray diffraction, elemental analysis, EPR, FTIR, and UV-Vis spectra, as well as cyclic voltammetry.

### 2.1. Description of the Crystal Structure of Cu(II) Complexes

**Figure 1:**
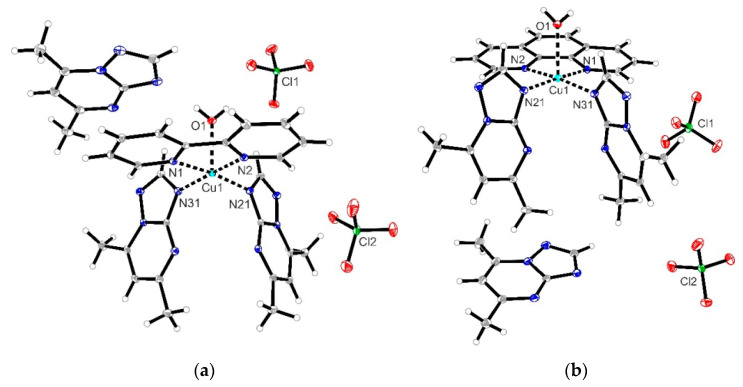
Crystal structures of compound (**1**) (**a**) and compound (**2**) (**b**). Thermal ellipsoids are set at 30% (for (**1**)) and 15% (for (**2**)) probability.

The structures of both complexes (**1**) and (**2**) were resolved by single-crystal X-ray diffraction, and a summary of crystallographic data is presented in Table 1. These compounds crystallize in the space group *P*-1 of the triclinic crystal system. The asymmetric unit consists of a cationic unit containing the Cu(II) ion, two perchlorate anions, and one uncoordinated 5,7-dimethyl-1,2,4-triazolo[1,5-*a*]pyrimidine (dmtp) ligand (Figure 1). For both complexes, the coordination geometry around the Cu(II) ion is square pyramidal, with the Cu(II) located in the basal plane as indicated by the continuous shape measure (CShM) values of 0.670 for (**1**) and 0.603 for (**2**) (Supplementary Table S1). Two corners of the basal plane are occupied by the bpy ligand, which coordinates in a chelate fashion with Cu–N bonds length of 2.031(2)/2.020(2) and 2.018(2)/2.038(2) Å, respectively. The other two corners of the basal plane are occupied by two unidentate dmtp ligands that generate Cu–N bonds with a length of 2.004(2)/1.989(2) and 1.991(2)/1.985(2) Å, respectively. A coordinated water molecule occupies the axial position of the square pyramidal surrounding with a Cu–O bond length of 2.233(2)/2.234(2) Å (Table 2).

**Figure 2:**
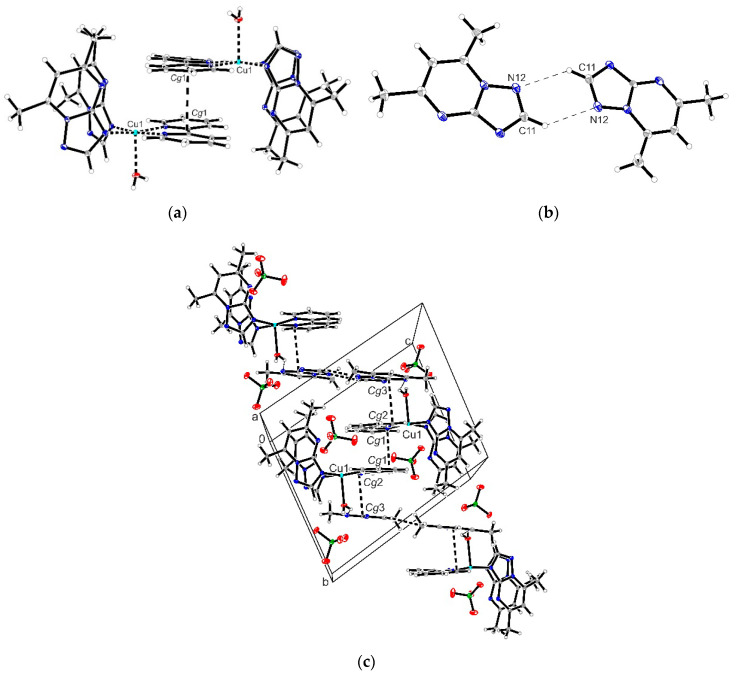
Dimer type formation between two cations of (**1**) through π⋯π interactions involving the bpy ligands (**a**); dimer type formation between two uncoordinated dmtp molecules involving CH⋯N interactions (**b**) and excerpt of the packing diagram of (**1**) presenting the formation of linear chains along the *b*-axis trough π⋯π, CH⋯N and OH⋯N interactions between the cations and the dmtp-free moieties (**c**).

Remarkably, both complexes crystallized with an additional uncoordinated dmtp ligand molecule in the asymmetric unit. The analysis of the packing diagram of both complexes reveals the formation of dimers between the cationic units and the free dmtp ligands. The dimers for compound (**1**) are shown in Figure 2, while those for compound (**2**) are presented in Supplementary Figures S1 and S2.

In the solid state, a linear chain along the *b*-axis involving alternating π⋯π interactions between the bpy ligands of two cations (*Cg*1⋯*Cg*1 3.377 Å) and π⋯π interactions between a part of the bpy ligand and the five-member aromatic ring of the uncoordinated dmtp molecule (*Cg*2⋯*Cg*3 3.465 Å) was observed for complex (**1**) (Figure 2, Supplementary Table S2). Similar interactions involving alternating π⋯π interactions between the phen ligands of two complex moieties (*Cg*1⋯*Cg*1 3.594 Å) and π⋯π interactions between a part of the phen and the five-member aromatic ring of the free dmtp (*Cg*2⋯*Cg*3 3.463 Å) can be noticed for complex (**2**) (Supplementary Figure S3, Supplementary Table S3).

### 2.2. Physico-Chemical Characterization of Complexes

#### 2.2.1. FT-IR Spectra

In the complexes spectra, the band associated with the triazolopyrimidine-fused rings (1632 and 1630 cm^−1^) is slightly shifted to lower wavenumbers in comparison with the free ligand (1635 cm^−1^; Supplementary Figure S4), as was observed for other complexes with tpds coordinated through the N^3^ atom [11]. The N-N-chelating heterocycle rings generate bands characteristic for overlapping ν(C=N) and ν(C=C) stretching vibrations in the 1440–1600 cm^−1^ range. Still, these bands display a low intensity along with that characteristic for a pyrimidine moiety. The intense bands at 1095/1099 cm^−1^ are assigned to ν_3_(ClO_4_), and the medium ones at 623/624 cm^−1^ are assigned to ν_4_(ClO_4_) stretching vibrations and account for perchlorate presence as a free ion [30]. The low−intensity bands at 417 and 432 cm^−1^ come from ν(Cu–N) stretching vibrations.

#### 2.2.2. UV-Vis Spectra

In the visible region of the diffuse reflectance spectra of the complexes, an unsymmetrical absorption band at 590/595 nm with a shoulder at higher wavenumbers (685/670 nm) can be noticed (Supplementary Figures S5 and S6). This aspect is characteristic of copper(II) ions in a distorted square pyramidal stereochemistry [31]. As a result of coordination, the band assigned to intraligand π→π* transitions for both ligands appears in the 260–340 nm range.

#### 2.2.3. EPR Spectroscopy

##### Solid-State EPR Spectroscopy

**Figure 3:**
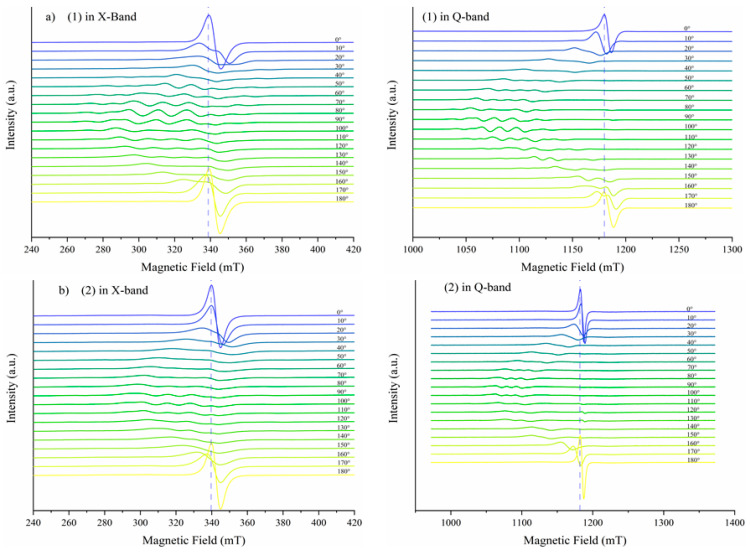
EPR spectra in X and Q-band with rotation along the main magnetic field of complex (**1**) (**a**) and complex (**2**) (**b**).

The EPR spectra obtained in the bc crystal plane at X- and Q-band and room temperature are shown in Figure 3. A single Lorentzian-shaped resonance line, characteristic of a strong exchange, was observed for a magnetic field orientation at 0 and 180° at both microwave frequencies, which is in good agreement with the results regarding the structure of the complexes where the Cu(II) is located in the basal plane of a square pyramid stereochemistry. In contrast, spectra with a resolved hyperfine structure, typical of weak exchange, were obtained for field orientations in the range of 20 to 160°. The g tensor for both complexes was an axial one with g_‖_ = 2.24 and g_⊥_ = 2.06 for complex (**1**) and g_‖_ = 2.25 and g_⊥_ = 2.057 for complex (**2**). The hyperfine coupling constants A_‖_ = 16.7 mT for complex (**1**) and A_‖_ = 14 mT for complex (**2**), respectively, were obtained. These values are in excellent agreement with those reported in the literature for Cu(II) in an axial distorted symmetry, such as the square pyramidal one [32].

##### Solution EPR Spectroscopy

**Figure 4:**
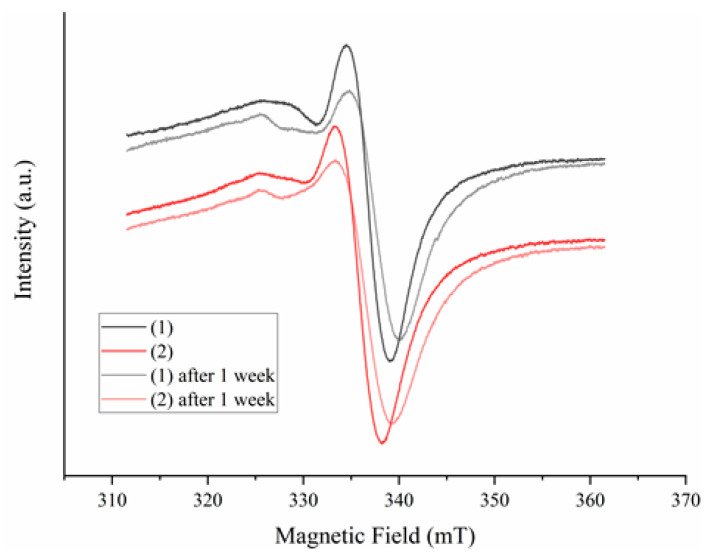
EPR spectra of complexes (**1**) and (**2**) in DMSO freshly prepared and after one week.

In order to determine their biological activity, the complexes were solubilized in dimethyl-sulfoxide (DMSO); therefore, their stability in this solvent was evaluated using EPR spectroscopy. For this purpose, the intensity and line shape of the EPR signal of 5 mM complex solutions were monitored over one week. As shown in Figure 4, no significant changes occurred after this period, indicating that the complexes are stable in DMSO.

##### Voltammetric Studies

The redox properties of the complexes were studied by cyclic voltammetry in order to obtain information concerning their oxidation potential, an important parameter that accounts for their interactions with redox active species in biological systems. The results were collected over two successive cycles in the anodic +0.15–+0.9 and, respectively, cathodic +0.9–−1.2 V, potential ranges at a 0.05 *V*/*s* scan rate were compared with those obtained for [Cu(DMSO)_6_](ClO_4_)_2_ under the same conditions (Table 3).

**Figure 5:**
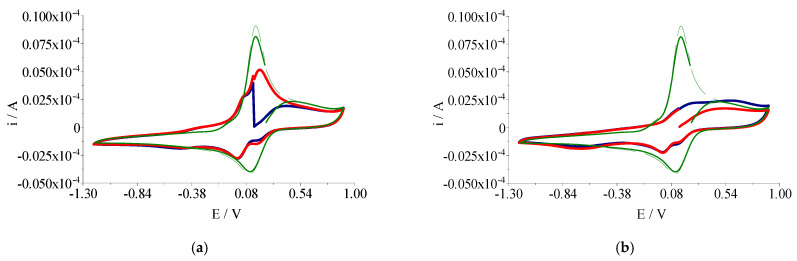
Cyclic voltammograms of complex (**1**) (**a**) and complex (**2**) (**b**), first cycle (blue line), second cycle (red line), cyclic voltamamograms of [Cu(DMSO)_6_](ClO_4_)_2_ (green line—first cycle and dashed green line—second cycle) in DMSO, (0.1 M Bu_4_NClO_4_; scan rate: 0.050 V/s, 0.3 mM, working electrode: platinum disk, reference electrode: Ag/AgCl (0.1 M Bu_4_NClO_4_ in DMSO).

The voltammograms for complexes (**1**) and (**2**) (Figure 5) display in the first cycle an oxidation step at ~+0.400 V assigned to Cu(II) to Cu(III) oxidation at E_pa1_ and three reduction waves corresponding to the sequences Cu(III)/Cu(II) at E_pc1_, Cu(II)/Cu(I) at E_pc2_ and Cu(I)/Cu(0) at E_pc3_, respectively (Table 3). The presence of these waves indicates that the organic ligands induce a higher stability of the Cu(III) and Cu(I) species in complexes (**1**) and (**2**) in comparison with [Cu(DMSO)_6_](ClO_4_)_2_.

The second cycle of the voltammograms shows the same peaks on the cathodic part and a single large one accompanied by a prepeak. In the case of complex (**2**) it is observed that all values corresponding to reduction processes are shifted to more negative values.

The value of the half-wave potentials versus Ag/AgCl (0.1 M Bu_4_NClO_4_ solution in DMSO) indicates values of Cu(III)/Cu(II) redox coupling included in the +0.3–+0.9 V range, a proper potential for hydrogen peroxide reduction by copper complexes [33].

### 2.3. Biological Characterization

#### 2.3.1. Cell Viability and Lactate Dehydrogenase Assays

Chemotherapy for melanoma that cannot be removed by surgery includes the use of some organic drugs like dacarbazine, cobimetinib fumarate, and trametinib, alone or in as a combined treatment [34]. However, their use is accompanied by severe side effects, including an increased risk of infection as well as resistance development. As a result, there is a continuous search for new antitumor drugs with reduced side effects and lower toxicity. The Cu(II) systems represent beneficial species from this point of view, considering their reduced systemic toxicity [16]. Thus, several Cu(II) complexes were studied and proved to be as effective for melanoma treatment [18,29,35,36,37,38,39,40,41,42,43]—some having, in addition, low toxicity on normal cells [18,29,39,40,41,42,43].

In order to assess cellular viability, the complexes and the dmtp ligand were tested against the B16 murine melanoma cell line, and data were compared with those obtained in the BJ normal cell line. The results for compound (**1**) reported in Figure 5A show that BJ cells were not affected over the range of tested concentrations, independent of the duration of the treatment. However, compound (**1**) reduced the viability of B16 cells—the effect being proportional with the tested concentration, both at 24 h and 48 h. The half-maximal inhibitory concentration (IC_50_) of compound (**1**) was 5.76 µg/mL (6.50 µM) at 24 h and 3.9 µg/mL (4.42 µM) at 48 h (Table 4). The results for compound (**2**) (Figure 5B) indicate that BJ cells treated for 24 h are affected by the compound, reducing their viability at the highest concentration by up to 45%, while for 48 h by up to 10%. The IC_50_ values obtained were 13.61 µg/mL (15.04 µM) for 24h treatment and 4.5 µg/mL (4.97 µM) for 48 h, respectively (Table 4). For B16 cells, when treated with (**2**), the cell viability decreased to 5%, with IC_50_ values of 6.88 µg/mL (7.6 µM) after 24 h treatment and 4.15 µg/mL (4.58 µM) after 48 h, respectively (Table 3). The ligand dmtp did not affect the viability of either BJ or B16 cells at 24 or 48 h.

It is worth mentioning that for other copper compounds tested on melanoma cells, the reported IC_50_ values were similar [35,36,37,38,39,40,41,42,43], and only one study reported lower values [38] compared with those found for complex (**1**).

A summary of IC_50_ data recorded at 48 h for B16 cells and TI values for perchlorate Cu(II) complexes with N-N [29] and tpds (dmtp and pmtp [18]) ligands is presented in Table 5. An improved activity of complex (**1**) in comparison with similar species bearing bpy and bpy/pmtp ligands was confirmed by the lowest value of 4.42 μM of IC_50_.

Based on the IC_50_ reported in Table 3, the therapeutic index (TI) was calculated; a higher TI value indicates a better cell selectivity. When no toxicity was detected at the highest concentration achieved in our experiments, a value two times higher was used for this calculation. Thus, from these data, it can be seen that compound (**1**) proved to be the most efficient against cancer cells, with a TI of 8.68 after 24 h and 12.82 at 48 h. For compound (**2**), the TI was 1.98 at 24 h and 1.08 at 48 h, respectively.

As a comparison, the TI values suggested a better activity of bpy derivatives in comparison with phen ones, and for dmtp species in comparison with pmtp. This could be related to a lower molar weight that allows an enhanced solubility, and also to the less bulky substituents that allow better interactions with the cellular targets.

**Figure 6:**
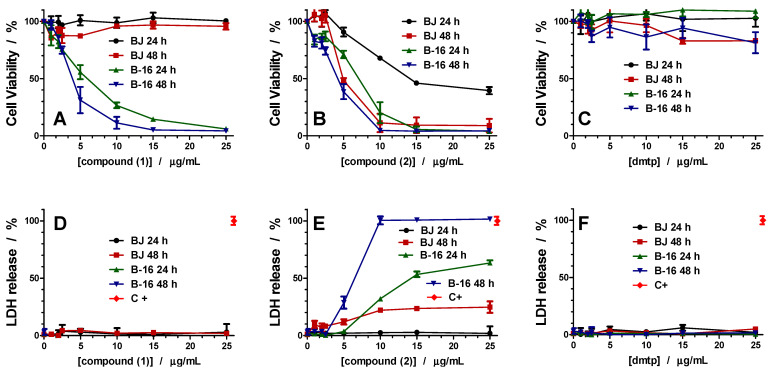
Cellular viability (**A**–**C**) and LDH release (**D**–**F**) of complexes (**1**), (**2**), and dmtp against BJ and B16 cells after 24 and 48 h.

To determine if the compounds affect membrane integrity, the lactate dehydrogenase (LDH) was evaluated at 24 h and 48 h (Figure 6D–F). Compound (**1**) did not affect the membrane integrity independent of the tested conditions (Figure 6D). When BJ cells were treated with compound (**2**) for 24 h, the membrane integrity was not affected (Figure 6E). However, at 48h, compound (**2**) induced an LDH release of around 20%. As for the B16 cells, compound (**2**) induced a 60% LDH release at 24 h and 100% LDH release at 48 h. These results indicate that compound (**2**) slightly affects the membrane integrity of BJ cells with increasing time and concentration. However, the effect is more severe for B16 tumoral cells. Similar to compound (**1**), dmtp did not affect the membrane integrity of the cells (Figure 6F).

#### 2.3.2. Cell Morphology

The morphological changes induced by the treatment of the BJ and B16 cell lines with the tested compounds at two different concentrations (2.5 and 10 µg/mL) applied for 24 h were investigated by fluorescence microscopy.

**Figure 7:**
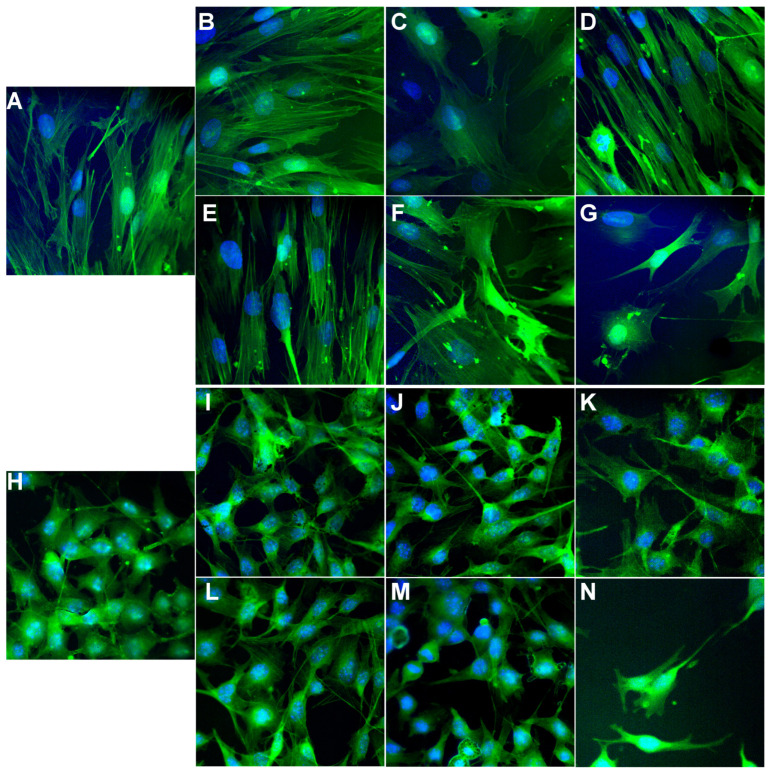
Fluorescence microscopy images recording morphological changes of cytoskeleton (BJ cells: control cells (**A**), treated with 2.5 µg/mL/16.89 µM (**B**) and 10 µg/mL/67.57 µM (**E**) dmtp, 2.5 µg/mL/2.83 µM (**C**) and 10 µg/mL/11.35 µM (**F**) compound (**1**) and 2.5 µg/mL/2.76 µM (**D**) and 10 µg/mL/11.05 µM (**G**) compound (**2**) for 24 h; B16 cells: control cells (**H**), treated with 2.5 µg/mL/16.89 µM (**I**) and 10 µg/mL/67.57 µM (**L**) dmtp, 2.5 µg/mL/2.83 µM (**J**) and 10 µg/mL/11.35 µM (**M**) compound (**1**) and 2.5 µg/mL/2.76 µM (**K**) and 10 µg/mL/11.05 µM (**N**) compound (**2**) for 24 h).

In BJ cells, dmtp at both concentrations (2.5 μg/mL/16.89 µM and 10 μg/mL/67.56 µM) did not alter their morphology (Figure 7B,E) compared with the control (Figure 6A). Similarly, no effect was observed for concentrations of 2.5 or 10 μg/mL (2.84 or 11.35 µM) in the case of compound (**1**) (Figure 7C,F). In the case of (**2**), only a concentration of 10 μg/mL (11.05 µM) affected the cellular morphology, by altering the cellular cytoskeleton (Figure 7G).

The morphology of B16 cells treated by dmtp, (**1**) and (**2**) for 24 h is not altered at a concentration of 2.84 μM and 2.76 μM, respectively (Figure 7) as compared with the control. In contrast, at a concentration of 10 μg/mL (11.05 µM), significant changes are observed for complex (**2**) (Figure 7N).

#### 2.3.3. Microbiological Assay

Usually, tumor development produces an immunosuppressive effect and gut microbiota dysbiosis, and thus, the development of infections associated with opportunistic microorganisms is favored. Thus, it is essential for compounds with antitumor activity to also possess an antimicrobial potential directed towards pathogenic species. As a result, the antimicrobial activity of complexes (**1**) and (**2**) was evaluated against Gram-negative (*E. coli* 25922, *P. aeruginosa* ATCC 27853) and Gram-positive (*S. aureus* ATCC 25853, MRSA 388) bacteria, frequently isolated from cancer patient infections.

The results showed that the compounds exhibited a more potent antimicrobial effect against the Gram-positive bacteria, enhanced in comparison with that of dmtp. Complex (**2**) exhibited the best antibacterial activity, with minimal inhibitory concentration (MIC) values ranging from 0.34 to 0.02 mM (Table 6). It is noteworthy that complex (**2**) exhibited strong antimicrobial activity against MRSA 388. MRSA has been mentioned in the EU/EEA (EARS-Net) Annual Epidemiological Report for 2019 as one of the most common causes of bloodstream infections, exhibiting a high burden in terms of morbidity and mortality [44]. These strains have become a severe clinical and epidemiological problem, as resistance to methicillin implies resistance to all β-lactam antibiotics [45].

The violet crystal quantification of biofilm formed on polystyrene surfaces in the presence of different concentrations of the complexes showed that both compounds were able to inhibit biofilm formation at the initial stage by reducing the expression of adhesion proteins, with the minimal biofilm eradication concentration (MBEC) values ranging from 0.71 to 0.04 mM (Table 7). The strongest antibiofilm effects were noticed against Gram-positive bacteria, including the MRSA strain. Methicillin resistance plays a crucial role in staphylococcal biofilm development, primarily mediated by the production of eDNA and cell surface adhesion proteins. Previous reports showed that Cu(II) complexes exhibit strong antimicrobial activity against both MRSA and methicillin-susceptible *Staphylococcus aureus* (MSSA) clinical isolates of both human and animal origin. It is not only effective in killing planktonic cells, but also has antimicrobial activity against biofilm and intracellular bacterial cells [46], therefore being a promising lead for the development of novel therapeutic strategies for persistent bacterial infections [47].

A comparison of MIC and MBEC data for perchlorate Cu(II) complexes with N-N and tp derivatives (dmtp and pmtp) ligands is presented in Table 8 and Table 9. From these data it can be concluded that the optimal activity of compound (**1**) (MIC of 0.18 mM) was observed in the case of the *S. aureus* strain, and for compound (**2**) in the case of the MRSA strain (MIC of 0.02 mM). Concerning the anti-biofilm activity, only complex (**2**) exhibited improved activity compared to similar species in interactions with the MRSA strain, as revealed by the MBEC value of 0.04 mM.

### 2.4. Complexes’ Interactions with ROS and DNA

The Cu(II) complexes’ interactions with DNA are complex and usually involve its degradation through reactive oxygen species (ROS) generated in Fenton-like reactions [33]. This interaction is favored when the ligands exhibit the ability to intercalate into the DNA strands. As a result, the complexes’ interactions with ROS and DNA were studied in order to propose a possible mechanism of action.

#### 2.4.1. Superoxide Scavenging Ability

**Figure 8:**
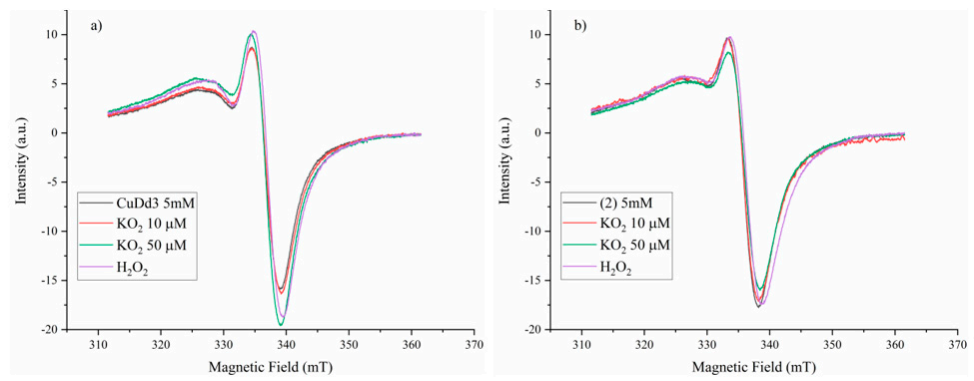
EPR spectra of complex (**1**) (**a**) and (**2**) (**b**) in interaction with O_2_^−^ and H_2_O_2_.

EPR spectroscopy was used to test the compounds’ ability to scavenge or trap reactive oxygen species. Figure 8 shows the EPR signal evolution as a function of the concentration of O_2_^−^ and H_2_O_2_ species added to a 5 mM DMSO complex solution. KO_2_ is highly reactive and was added in 10 and 50 μM concentrations. Complex (**2**) showed some scavenging ability towards the O_2·_^−^ radicals, which was demonstrated by the decrease in the EPR signal intensity when adding KO_2_. When adding H_2_O_2_ as an OH∙ radical source, the EPR signal remained unchanged, indicating no reaction towards the hydroxyl radicals. The intensity of the EPR spectra increased at both KO_2_ and H_2_O_2_, indicating that the superoxide species and hydroxyl radicals were trapped rather than scavenged by complex (**1**).

#### 2.4.2. Effect of Compounds on *Saccharomyces cerevisiae* Cells

As complexes (**1**) and particularly (**2**) exhibited some superoxide-scavenging activity, as seen by EPR spectroscopy (Figure 8), we checked if the two compounds had any protective action against the deleterious effect of superoxide ions in vivo. To check this possibility, compounds (**1**) and (**2**) were tested on *Saccharomyces cerevisiae* strains defective in cytosolic Cu,Zn-superoxide dismutase SOD1 (*sod1Δ* strain) or mitochondrial Mn-superoxide dismutase SOD2 (*sod2Δ* strain). Both *sod1Δ* and *sod2Δ* strains are sensitive to superoxide-generating drugs, such as menadione or paraquat. We noted that the growth of yeast cells—both wild type (WT) and knockout strains *sod1Δ* or *sod2Δ—*was not affected by exposure to compounds (**1**) and (**2**), and this apparent lack of toxicity was recorded for concentrations up to 2 mM in the cell environment. Nevertheless, we noticed that both (**1**) and (**2**) alleviated the sensitivity of *sod1Δ* and *sod2Δ* to menadione (a superoxide-generating drug; Figure 8a), indicating that when present in non-toxic, physiologic concentrations, compounds (**1**) and **(2**) can act as superoxide scavengers in yeast cells devoid of regular SOD activity.

**Figure 9:**
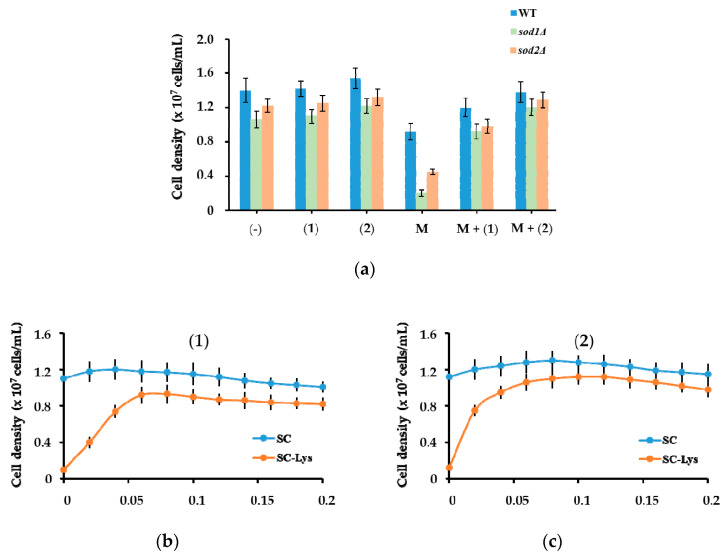
Effect of (**1**) and (**2**) on alleviating superoxide-related growth defects in *Saccharomyces cerevisiae*. Effect on cell growth in the presence of the superoxide-generating drug, menadione (**a**). Yeast cells were inoculated in SC (10^6^ cells/mL), and their proliferation was determined spectrophotometrically (OD_660_) after 16 h of exposure to 0.1 mM of each of the indicated compounds. M, menadione. Effect of compounds (**1**)/(**b**) and (**2**)/(**c**) on alleviating lysine auxotrophy of *sod1Δ*. Mid-log phase *sod1Δ* cells suspended (1 × 10^6^ cells/mL) in SC or SC-Lys were grown for 16 h (30 °C, 250 rpm) in the presence of various concentrations of the complex before cell growth was determined spectrophotometrically (OD_660_). Values are mean ± SD of duplicate determinations of three biological replicates.

Deleting the *SOD1* gene in yeast is accompanied by lysine auxotrophy, apparently caused by superoxide generation under aerobic conditions, affecting the metabolic pathway involved in lysine biosynthesis [48]. This phenotype affords a straightforward approach for testing the superoxide-scavenging traits of various chemicals [49,50]. We, therefore, tested whether our compounds alleviate lysine auxotrophy of *sod1Δ* cells. We found indeed that (**1**), and to a higher extent (**2**), improved the growth of *sod1Δ* mutants in SC-Lys medium (Figure 9b,c).

#### 2.4.3. DNA-Binding Properties of Compounds

Complexes of (**1**) and (**2**) exhibited low toxicity against eukaryotic cells and even showed a protective effect against the deleterious action of superoxide ions (Figure 8). Despite their apparent low toxicity, we further investigated the interactions between complex (**1**) or (**2**) with DNA. The modifications induced by DNA to the spectral characteristics of the two complexes were determined through both absorption and fluorescence spectra modifications.

##### Absorption Spectra

Absorption spectroscopy is often used to monitor the interactions of a compound with DNA through band intensity modification, which generally indicates intercalative abilities [51]. The binding of complexes (**1**) and (**2**) to λ-DNA was determined by monitoring the absorption spectra of a fixed complex concentration (5 µM), to which an increasing amount of DNA solution was added. The binding of complex (**1**) to λ-DNA led to a decrease in the absorption intensity of the band (Figure 9a), indicating possible intercalation of (**1**) into λ-DNA strands.

**Figure 10:**
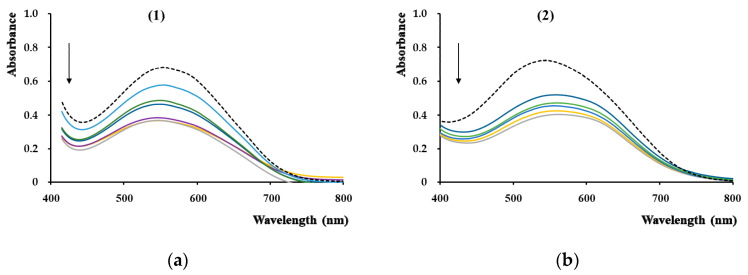
Absorption spectra of (**1**) (**a**) and (**2**) (**b**) in the absence of DNA (black, dotted line) or in Table 5. µM complex concentration.

The binding of complex (**2**) to λ-DNA was also accompanied by a decrease in the absorption intensity of the spectral bands, with DNA concentrations increasing (Figure 10b). The hypochromism observed for the absorption band indicates that similarly to (**1**), complex (**2**) also binds to λ-DNA, probably by intercalation through the planar aromatic rings of the N-N-chelating ligands. Continuing to increase λ-DNA after reaching saturation was no longer accompanied by the hypochromic effect, and the spectra were not significantly different after reaching saturation.

##### Fluorescence Spectra

**Figure 11:**
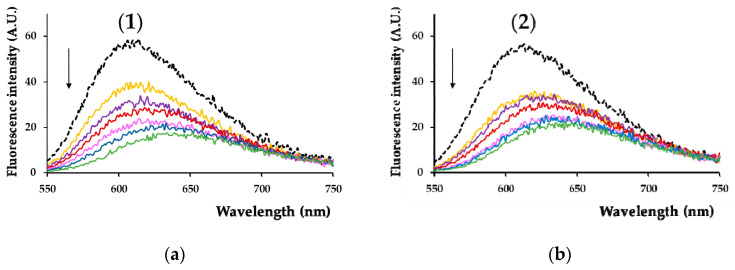
Emission spectra of λ-DNA/EB in Tris/HCl/NaCl buffer, pH = 8, in the absence (black curves) or the presence of increasing concentrations of (**1**) (**a**) and (**2**) (**b**). Arrows indicate the increase in [complex].

The capacity of (**1**) and (**2**) to quench the fluorescence of the λ-DNA/EB adduct was also determined. Ethidium bromide (EB) is one of the most utilized dyes for DNA detection as it causes intense fluorescence after intercalating into DNA [52]. We noticed that when adding increments of (**1**) and (**2**) to λ-DNA pre-treated with EB, the fluorescence intensity decreased—a clear indication that the complex competes with EB to the binding sites of DNA (Figure 11).

#### 2.4.4. Nuclease Activity

**Figure 12:**
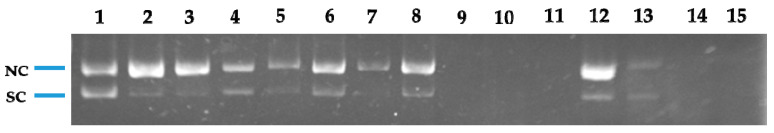
Gel electrophoresis image of pRSII425 (100 ng/μL) after incubation for 1 h at 37 °C with the indicated compounds. Lane 1: pRSII325 alone; Lane 2: pRSII325 + 1 mM ascorbate; Lane 3: pRSII325 + 1 mM H_2_O_2_; Lane 4: pRSII325 + 0.1 mM (**1**); Lane 5: pRSII325 + 0.5 mM (**1**); Lane 6: pRSII325 + 0.1 mM (**2**); Lane 7: pRSII325 + 0.5 mM (**2**); Lane 8: pRSII325 + 0.1 mM (**1**) + 1 mM ascorbate; Lane 9: pRSII325 + 0.5 mM (**1**) + 1 mM ascorbate; Lane 10: pRSII325 + 0.1 mM (**1**) + 1 mM H_2_O_2_; Lane 11: pRSII325 + 0.5 mM (**1**) + 1 mM H_2_O_2_; Lane 12: pRSII325 + 0.1 mM (**2**) + 1 mM ascorbate; Lane 13: pRSII325 + 0.5 mM (**2**) + 1 mM ascorbate; Lane 14: pRSII325 + 0.1 mM (**2**) + 1 mM H_2_O_2_; Lane 15: pRSII325 + 0.5 mM (**2**) + 1 mM H_2_O_2_.

To investigate the DNA cleavage activity of the two Cu(II) complexes, plasmidial DNA was incubated with (**1**) and (**2**), both in the absence or in the presence of activating agents, such as H_2_O_2_ or ascorbate. Exposure of DNA to the Cu(II) complexes in the presence of H_2_O_2_ exploits the CuII/III redox couple, while in the presence of ascorbate, the CuI/II redox couple is exploited [53]. The nuclease activity of copper(II) complexes (**1**) and (**2**) was tested on plasmid pRSII325, which is a 6835 bp (base pair) circular plasmid. Gel electrophoresis of purified pRSII325 identified two main bands belonging to the supercoiled (SC) plasmid, which migrates faster, and the nicked-circular (NC) plasmid, which migrates slower (Figure 12, lane 1).

It was noted that both ascorbate (Figure 12, lane 2) and H_2_O_2_ (Figure 12, lane 3) alone caused DNA relaxation to the NC form. Compounds (**1**) and (**2**) alone also caused plasmid relaxation to the NC form in a dose-dependent manner (Figure 12, lanes 4–7). Incubating pRSII325 with (**1**) in the presence of either ascorbate (Figure 12, lanes 8 and 9) or H_2_O_2_ (Figure 12, lanes 10 and 11) led to DNA degradation, indicating a strong nuclease activity—especially in the presence of H_2_O_2_. The nuclease activity of (**2**) followed a pattern similar to (**1**), with H_2_O_2_ strongly enhancing the nuclease activity, apparently irrespective of (**2**) concentration (Figure 12, lanes 14 and 15). Otherwise, the complexes’ ability to interact with H_2_O_2_ was evidenced by EPR and cyclic voltammetry experiments. As in the case of (**1**), a higher concentration of (**2**) was needed for full nuclease activity enhanced by ascorbate (Figure 12, lanes 12 and 13).

## 3. Materials and Methods

### 3.1. Reagents

The chemicals for the synthesis of the complexes were purchased from Sigma-Aldrich (Darmstadt, Germany) (copper(II) perchlorate hexahydrate (≥99.99% trace metals basis), 2,2′-biyridine (bpy, 99%), 1,10-phenantroline (phen, 99%), 2,3-pentanedione (97%) and 3-amino-4H-1,2,4-triazole (96%)) and Merck (Darmstadt, Germany; dibenzo-18-crown-6-ether, potassium superoxide) at reagent grade and were used as received, without further purification. The 5,7-dimethyl-1,2,4-triazolo[1,5-*a*]pyrimidine (dmtp) was synthesized by [1+1} condensation of 3-amino-4H-1,2,4-triazole and 2,3-pentanedione.

### 3.2. Physical Measurements

A EuroEA elemental analyzer (Perkin Elmer, Waltham, MA, USA) was used for chemical analyses (C, N, and H). Fourier Transform Infrared spectroscopy (FTIR) spectra were recorded in KBr pellets with a Tensor 37 spectrometer (Bruker, Billerica, MA, USA) in the 400–4000 cm^−1^ range. UV-Vis spectroscopy was performed in the solid state on a V 670 spectrophotometer (Jasco, Easton, MD, USA) with Spectralon as standard in the 200–1500 nm range. The X-band Electron Paramagnetic Resonance (EPR) spectroscopy measurements were carried out with a continuous wave X-Band EMX plus EPR spectrometer (Bruker AXS GmbH, Karlsruhe, Germany) equipped with a Bruker X-SHQ 4119HS-W1 X-Band resonator. The measurement parameters for the X-Band measurements, if not otherwise mentioned, were set as follows: microwave frequency 9.879 GHz, microwave power 2 mW, modulation amplitude 0.1 mT, conversion time 26.67 ms, time constant 10.24 ms at one scan. A continuous-wave Q-band Bruker ELEXSYS E500Q EPR spectrometer and an ER 5106 QT-W resonator were used for the Q-Band measurements. The measurement parameters for the Q-Band measurements, if not otherwise mentioned, were set as follows: microwave frequency 34.1507 GHz, microwave power 0.59 mW, modulation amplitude 0.1 mT, conversion time 40 ms, time constant 20.48 ms at one scan. All measurements were carried out at room temperature. Cyclic voltammograms were recorded at room temperature with an electrochemical system (potentiostat/galvanostat) Autolab PGSTAT 12 using the GPES (General Purpose Electrochemical System) software. These studies were carried out in inert atmosphere (Ar 99.9999%) in DMSO containing tetrabutylammonium perchlorate (Bu_4_NClO_4_) 0.1 M as supporting electrolyte. The reference electrode was Ag/AgCl separated from the solution by a bridge filled with a 0.1 M Bu_4_NClO_4_ solution in DMSO. The counter electrode was the platinum wire. A platinum disk electrode with 0.3 mm diameter was used as working electrode. The complexes concentration was 0.3 mM in all measurements.

X-ray single crystal diffraction data sets for (**1**) were collected with a Bruker APEX II CCD diffractometer (Bruker AXS, Karlsruhe, Germany). Programs used: data collection: *APEX3 V2016.1-0* [54]; cell refinement: *SAINT V8.37A* [54]; data reduction: *SAINT V8.37A* [54]; absorption correction, *SADABS V2014/7* [54]; structure solution *SHELXT-2015* [55]; structure refinement *SHELXL-2015* [56]. For compound (**2**), data sets were collected with a Nonius Kappa CCD diffractometer (Bruker Nonius B.V., Delft, Holland). Programs used: data collection, *COLLECT* [57]; data reduction Denzo-SMN [58]; absorption correction *Denzo* [59]; structure solution *SHELXT-2015* [55]; structure refinement *SHELXL-2015* [56] and graphics, *XP* [60]. *R*-values are given for observed reflections, and *w*R^2^ values are given for all reflections.

Exceptions and special features: For compound (**2**), one perchlorate anion was found to be disordered over two positions in the asymmetric unit. Several restraints (SADI, SAME, ISOR, and SIMU) were used to improve refinement stability. Additionally, for compounds (**1**) and (**2**), two badly disordered water molecules were found in the asymmetrical unit and could not be satisfactorily refined. The *SQUEEZE* program [61] was therefore used to remove the effects of the solvent mathematically. The quoted formula and derived parameters do not include the squeezed solvent molecules. The crystallographic data for complexes (**1**) and (**2**) were deposited at the Cambridge Crystallographic Data Centre as a supplementary publication with CCDC numbers 2067846 and 2067847, respectively. These data can be obtained free of charge via http://www.ccdc.cam.ac.uk/data_request/cif (accessed on 29 August 2021), by e-mailing data_request@ccdc.cam.ac.uk, or by contacting the Cambridge Crystallographic Data Centre, 12 Union Road, Cambridge CB2 1EZ, UK; Fax: +44-1223-336033.

### 3.3. Synthesis and Characterization of the Complexes

[Cu(dpy)(dmtp)_2_(OH_2_)](ClO_4_)_2_·dmtp (**1**): To a solution, containing copper(II) perchlorate hexahydrate (0.186 g, 0.5 mmol) in 50 mL ethanol, a solution containing dpy (0.078 g, 0.5 mmol) in 25 mL ethanol was added. This mixture was magnetically stirred at 50 °C for 2 h, until the color turned blue, and then a solution containing dmtp (0.222 g, 1.5 mmol) in 50 mL ethanol was added. The stirring of the reaction mixture continued for 4 h, while the blue color intensified. Crystals suitable for X-ray analysis were obtained after three weeks by slow evaporation of this solution. Yield: 78% (0.34 g). Analysis, found: C, 42.37; H, 3.75; N, 22.31%; calculated forCuC_31_H_34_N_14_O_9_Cl_2_ (M_w_: 881.15 g mol^−1^): C, 42.26; H, 3.89; N, 22.25%, IR (KBr pellet, cm^−1^): υ(H_2_O), 3511 w; υ(CH), 3136 w, 3112 w, 3083 w; υ(C=N)_trp_, 1632 m; υ(C=N)_pym_, 1556 m; υ(C=N)+υ(C=C), 1602m, 1496m; υ_3_(ClO_4_), 1095 vs; υ_4_(ClO_4_), 623 m; υ(Cu-N), 417 w, UV-Vis (solid, nm): π → π*, 290, 345; d_xz_, d_yz_ → d_z2_, 590; d_xy_ → d_z2_, 675.

[Cu(phen)(dmtp)_2_(OH_2_)](ClO_4_)_2_·dmtp (**2**): To a solution, containing copper(II) perchlorate hexahydrate (0.186 g, 0.5 mmol) in 50 mL water, a solution containing phen (0.090 g, 1 mmol) in 25 mL ethanol was added. This mixture was magnetically stirred at 50 °C for 2 h, until the color turned green, and then a solution containing dmtp (0.222 g, 1.5 mmol) in 25 mL ethanol was added. The stirring of the reaction mixture continued for 4 h, until the blue color was formed. Crystals suitable for X-ray analysis were obtained after two weeks by slow evaporation of this solution. Yield: 75% (0.34 g). Analysis, found: C, 43.83; H, 3.75; N, 21.68%; calculated forCuC_33_H_34_N_14_O_9_Cl_2_ (M_w_: 905.17 g mol^−1^): C, 43.79; H, 3.79; N, 21.66%, IR (KBr pellet, cm^−1^): υ(H_2_O), 3517 w; υ(CH), 3137 w, 3110 w, 3078 w; υ(C=N)_trp_, 1630 m; υ(C=N)_pym_, 1553 m; υ(C=N)+υ(C=C), 1474 m, 1443 m; υ_3_(ClO_4_), 1099 vs; υ_4_(ClO_4_), 624 m; υ(Cu-N), 432 w, UV-Vis (solid, nm): π → π*, 265, 360; d_xz_, d_yz_ →d_z2_, 610; d_xy_ → d_z2_, 680.

### 3.4. Biological Characterization of Compounds

#### Screening of the Antibacterial Properties

The antibacterial assays were carried out using two Gram-negative (*Escherichia coli* ATCC 25922, *Pseudomonas aeruginosa* ATCC 27853) and two Gram-positive (*Staphylococcus aureus* ATCC 25923, MRSA (meticillin-Resistant *S. aureus*) 388 (ATCC, American Type Culture Collection) bacteria.

The antimicrobial activity of the two complexes (**1**) and (**2**) was assessed using their minimum inhibitory concentration (MIC) by the broth microdilution method, as previously described by the Clinical and Laboratory Standard Institute (CLSI; M07-A9 document) [62]. A broth microdilution assay was performed in sterile 96-well plates using Muller-Hinton broth (Scharlau; MHB). The compounds solubilized in DSO (1,000 μg/mL) were serially diluted in 90 μL MHB, in concentrations ranging between 500 and 0.97 μg/mL. Overnight cultures were used to prepare suspension in phosphate-buffered saline (pH 7.0) to match a 0.5 McFarland density. The bacterial suspensions were further diluted to 1:100 by adding 0.1 mL of bacterial suspension to 9.9 mL of MHB. A volume of 10 μL of this dilution was added to each well of columns 1–10, containing different concentrations of the tested compounds. This resulted in the final desired inoculum of 105 cfu/mL. Additionally, 10 μL of the diluted bacterial suspension was added in column 11 (the growth control). A volume of 100 μL MHB was added to the microtiter plates’ sterility control wells (column 12). After inoculation, the plates were incubated at 35 ± 2 ºC for 18 h. Growth was measured as light absorbance (620 nm) in comparison to an uninoculated well (negative control). It was detected using a microtiter plate reader (Apollo LB 911ELISA (Berthold Technologies GmbH & Co. KG, Waltham, MA, USA)). The MIC of each compound was defined as the lowest concentration required to inhibit bacterial growth.

The method for assessment of biofilm formation on polystyrene microtiter plates was based on previously published protocols [18,63,64]. The absorbance at 490 nm was measured with an Apollo LB 911ELISA (Berthold Technologies GmbH & Co. KG, Waltham, MA, USA) reader. The minimal biofilm-eradication concentration (MBEC) was defined as the lowest concentration of antibiotics required to eradicate the biofilm. All experiments were performed in triplicate.

### 3.5. In Vitro Cytotoxicity Assay

#### 3.5.1. Cell Culture Conditions

Human fibroblast cells (BJ—ATCC CRL-2522, USA) were grown in minimal essential medium (MEM) supplemented with 2 mM L-Glutamine, 10% fetal calf serum (FCS), 100 units/mL of penicillin, and 100 µg/mL of streptomycin at 37 °C in a humidified incubator under an atmosphere containing 5% CO_2_. Mouse melanoma cells (B16—ATCC CRL—6475, USA) were grown in DMEM (Dulbecco’s Modified Eagle Medium) supplemented with similar reagents as MEM. All cell cultivation media and reagents were purchased from Biochrom AG (Berlin, Germany) and Sigma-Aldrich (Darmstadt, Germany).

#### 3.5.2. Cell Viability Assay

Cell viability was evaluated using 3-(4,5-dimethylthiazol-2-yl)-2,5-diphenyltetrazolium bromide (MTT) assay as described previously [18]. First, the cells were seeded in 96-well plates (20000 cells/well) and cultured for 24 h in medium. After overnight incubation, the medium was changed, and the investigated sample at a concentration varying from 0.03 to 0.25 mg/mL was added for 24 h. The negative control was represented by cells cultivated in a medium without the investigated compounds. Following incubation, the medium was changed, and the MTT solution was added to each well to a final concentration of 1 mg/mL and incubated for an additional 4 h, at 37 °C. Finally, the medium was collected, and DMSO was used to dissolve the insoluble formazan product. The absorbance of the samples was recorded at 570 nm using a plate reader Mithras 940 (Berthold). The data were corrected for the background, and the percentage of viable cells was obtained using the equation:

Cell viability = [(A_570_ of treated cells)/(A_570_ of untreated cells)] × 100% (1)

The half-maximal inhibitory concentration (IC_50_) was determined by fitting the data with a sigmoidal logistical equation using the software Origin 8.1 (Microcal Inc., Los Angeles, CA, USA).

#### 3.5.3. Lactate Dehydrogenase (LDH) Release Assay

Membrane integrity after peptide treatment was assessed based on LDH release using a CytoTox 96 Non-Radioactive Cytotoxicity Assay (Promega), as previously described [65]. Cells were treated with different concentrations for 24 h, and the medium was used for the LDH assay. Maximal LDH release was obtained by complete cell lysis induced by using 1% of Triton X-100. The absorption resulting from LDH activity was measured in a microplate reader Mithras 940 (Berthold) at 490 nm, and the % LDH was calculated as: [(corrected absorbance of the LDH released in treated cells)/(corrected absorbance of the total LDH released)] * 100%.

#### 3.5.4. Phalloidin Staining and Cell Imaging

According to the manufacturer protocol, the cytoskeleton actin filaments of BJ and B16 cells were stained with phalloidin-FITC (Sigma-Aldrich, St. Louis, MO, USA). Briefly, cells were washed with PBS (5 min, 3 times), fixed for 5 min with 3% paraformaldehyde, washed three times with PBS, permeabilized with 0.1% Triton X-100 in PBS for 15 min, washed three times with PBS, stained with 20 μg/mL phalloidin-FITC at room temperature for 1 h and rewashed three times with PBS. The cell nucleus was stained with 8 μM of Hoechst 33342 solution for 10 min, washed three times with PBS, and finally, mounted and sealed on glass slides with FluorSave™ Reagent (Merck Millipore, Germany). The fluorescence images were acquired using an Andor DSD2 Confocal Unit (Andor, Ireland), mounted on an epifluorescence microscope, Olympus BX-51 (Olympus, Germany), equipped with a 40x and 100x objective and an appropriate DAPI/Hoechst filter cube (excitation filter 390/40 m, dichroic mirror 405 nm and emission filter 452/45 nm) and GFP/FITC filter cube (excitation filter 466/40 nm, dichroic mirror 488 nm and emission filter 525/54 nm).

#### 3.5.5. Superoxide Scavenging Ability

The superoxide scavenging ability of the complexes was tested using the KO_2_ compound as a superoxide source combined with EPR spectroscopy. To carry out the experiments, a 10 µM in DMSO solution of the complexes was mixed with different concentrations of KO_2_ in DMSO solution, and the EPR signal intensity changes were monitored. The KO_2_ was dissolved by complexation with dibenzo-18-crown-6-ether.

#### 3.5.6. Yeast Cells Experiments

The *Saccharomyces cerevisiae* laboratory strain BY4741 (*MAT*a; *his3Δ1*; *leu2Δ0*; *met15Δ0*; *ura3Δ0*) considered wild type (WT), and the isogenic strains *sod1Δ* and *sod2Δ* lacking the genes *SOD1* (encoding Cu/Zn-superoxide dismutase) and *SOD2* (encoding Mn-superoxide dismutase) were obtained from EUROSCARF (Frankfurt, Germany). Cells were maintained, manipulated and grown in a rich medium (YPD, 1% *w*/*v* yeast extract, 2% *w*/*v* peptone, 2% *w*/*v* glucose; 2% *w*/*v* agar was added for solid media) or on synthetic complete medium (SC, 0.67% *w*/*v* yeast nitrogen base without amino acids, 2% *w*/*v* glucose) supplemented with the necessary amino acids [66]. Lysine drop-out medium (SC-Lys) was prepared similarly to SC, omitting lysine from the recipe. We added the complexes to autoclaved media from DMSO sterile stocks (10 mM). An overnight culture was 10^3^-fold diluted in fresh medium and incubated (200 rpm, 30 °C) until the culture reached a density of 5 × 10^5^ cells. The compounds were added to the desired concentrations, and culture growth was monitored at 660 nm [67] in a 96-well multi-scanner plate reader equipped with a thermostat and shaker (Varioskan, Thermo Fischer Scientific, Vantaa, Finland). Three biological replicates were set for all determinations, and data were presented as average ± standard error (SE). The *Saccharomyces cerevisiae* laboratory strain BY4741 (*MATa*; *his3Δ1*; *leu2Δ0*; *met15Δ0*; *ura3Δ0*) considered wild type (WT), and the isogenic strains *sod1Δ* and *sod2Δ* lacking the genes *SOD1* (encoding Cu/Zn-superoxide dismutase) and *SOD2* (encoding Mn-superoxide dismutase) were purchased from EUROSCARF (Frankfurt, Germany). Cells were maintained and manipulated and grown in YPD medium (1% *w*/*v* yeast extract, 2% *w*/*v* peptone, 2% *w*/*v* glucose; 2% *w*/*v* agar was added for solid media) [66]. Complexes were added to autoclaved media from DMSO sterile stocks (10 mM). To determine the effect of compounds on yeast growth, a fresh overnight pre-culture was 10^3^-fold diluted in YPD and incubated (200 rpm, 30 °C) until the culture reached a density of 5 × 10^5^ cells/mL. The compounds were added at the desired concentrations, and cell proliferation was monitored at 660 nm [60] in a 96-well multi-scanner auto reader plate reader equipped with a thermostat and shaker (Varioskan Thermo Fischer Scientific, Vantaa, Finland). All determinations were done on three biological replicates.

#### 3.5.7. Spectroscopic Determination of the Compounds’ Interactions with DNA

To determine if the compounds can interact with DNA, the 48,500 base-pairs genomic DNA from bacteriophage lambda (λ-DNA, Promega, MA, USA) was used. A stock solution of 10 µM λ-DNA (base-pair concentration) was prepared in 10 mMTris/HCl buffer containing 1 mM NaCl, pH 8.

#### 3.5.8. Effect of DNA on Spectral Characteristics of Compounds

To determine the DNA intercalating ability of complexes (**1**) and (**2**), we used the 48,500 base-pairs genomic DNA from the bacteriophage lambda (λ-DNA, Promega, Madison, USA). A 10 µM λ-DNA (base-pair concentration) stock solution was prepared in 10 mM Tris/HCl buffer, pH 8, containing 1 mM NaCl. The UV-visible absorption titration experiments were done using a constant complex concentration (5 µM). The concentrations of the λ-DNA solutions were gradually increased until a saturation state was achieved [68,69]. After DNA addition, the solutions were left to equilibrate for 5 min at room temperature before recording the spectra with a Jasco V-630 spectrophotometer. Stock solutions of compounds (**1**) and (**2**) were prepared in DMSO (1 mM) and diluted to 5 µM in 10 mM Tris/HCl containing 1 mM NaCl, pH 8.

The UV-Vis absorption titration experiments were done, keeping the complex concentration constant (3 µM) and increasing the concentration of λ-DNA in Tris-HCl/NaCl buffer solution at room temperature until a saturation state was achieved [63,64]. After DNA addition, the solutions were allowed to equilibrate for 5 min at room temperature. The UV-Vis absorption spectra were recorded with a Jasco V-630 spectrophotometer, using a 10 mm quartz cell. Stock solutions of compounds were prepared in DMSO (1 mM) and diluted to 3 µM in 10 mMTris/HCl containing 1 mM NaCl, pH 8.

#### 3.5.9. Effect of Compounds on the Fluorescence of λ-DNA/Ethidium Bromide Adduct

The binding of complexes (**1**) and (**2**) to DNA was assayed by monitoring the quenching of fluorescence emitted by λ-DNA/ethidium bromide (EB) adduct (λ_excit_ = 510 nm, maximum emission at λ_em_ = 601 nm) in the presence of different concentrations of the tested compounds [68,69]. The fluorescence spectra were recorded in the range of 550–750 nm using a Thermo Fischer Scientific Varioskan Flash spectral scanning multimode reader (Vantaa, Finland). The spectra were recorded in suitable plates using 5 nm excitation and emission slits for all measurements.

The binding of compounds to λ-DNA was assayed by monitoring the quenching of fluorescence emitted by λ-DNA/ethidium bromide (EB) adduct (λ_excit_ = 510, maximum emission at λ_em_ = 601). The λ-DNA solution was added to the EB solution, prepared in the same buffer, then increasing concentrations of compounds were added to EB-DNA [52,70]. The fluorescence spectra were recorded in the range of 550–750 nm using a Thermo Fischer Scientific Varioskan Flash spectral scanning multimode reader (Vantaa, Finland). The spectra were recorded in suitable plates using 5 nm excitation and emission slits for all measurements.

#### 3.5.10. Nuclease Activity Assay

The nuclease activity of the compounds was determined using plasmid pRS325II DNA, which was a gift from Steven Haase (Addgene plasmid #35467, 6835 base pairs) [71]. The plasmid was amplified in *Escherichia coli* by transforming One Shot^®^ TOP10 chemically competent *E. coli* (Invitrogen, Thermo Fisher Scientific). The plasmid was isolated from positive colonies using a PureLink™ HiPure Plasmid Miniprep Kit (Invitrogen, Thermo Fisher Scientific). Plasmid (200 ng/sample) was exposed to complexes (**1**) and (**2**) in the presence of 1 mM hydrogen peroxide (to exploit the Cu(II/III) redox couple) or 1 mM ascorbic acid (to exploit the Cu(I/II) redox couple) [46]. The DNA cleavage experiments were done in a 9/1 (*v*/*v*) ratio of 50 mM Tris-HCl, pH 8, and DMSO. The samples were incubated for 1 h at 37 °C before a bromophenol blue/xylene cyanol-based loading dye (Roth, Germany) was added. The samples were loaded onto a 1% (*w*/*v*) agarose gel containing 1 μg/mL EtBr in 1 × TBE (Tris–boric acid–EDTA) buffer. Electrophoresis was performed at 50 V for 60 min in 1 × TBE buffer. The images of the fluorescent ethidium bromide-stained gels were captured using a gel documentation system (Doc-Print II, VilberLourmat, France). The cleavage experiments were done three times, with similar results. One representative gel is shown.

## 4. Conclusions

A novel series of copper(II) complexes with mixed ligands (5,7-dimethyl-1,2,4-triazolo[1,5-*a*]pyrimidine and 2,2′-bipyridine/1,10-phenanthroline) were stepwise synthesized, and structurally characterized. A complex supramolecular network is generated through π-π stacking interactions realized between both 2,2′-bipyridine/1,10-phenanthroline belonging to different cationic units as well as between these and free triazolopyrimidine moieties. Complex (**1**) exhibits the ability to reduce the viability of B16 cells in a dose-dependent manner and furthermore does not show any significant toxicity against BJ and *Saccharomyces cerevisiae* cells in the concentration ranges proven for the manifestation of biological activity. The value of the therapeutic index value and morphological modifications indicate that complex (**1**) was more susceptible than (**2**). Both complexes exhibited improved antimicrobial activity, as compared to the triazolopyrimidine ligand. Complex (**2**) was by far more active than (**1**) against the Gram-positive strains, susceptible or resistant, growing in planktonic or in biofilm states. Both MIC and MBEC values decreased in the case of complex (**2**) by 4 to 17 times compared to (**1**). The results suggest that both the antitumor and antimicrobial activity of these complexes involves intercalation into DNA strands as well as its damage through metallonuclease activity. Nuclease-like activity is enhanced in the presence of hydrogen peroxide, the complexes’ interactions with this ROS also being proved by EPR and cyclic voltammetry data. Overall studies indicate the antitumor potential of complex (**1**) and the antibacterial potential of complex (**2**), especially against the Gram positive strains. As a result, these species will be further studied in order to improve their solubility, and consequently their activity, by their inglobation in organic matrix.

## Figures and Tables

**Table 1:** Crystal data and structure refinement for compounds (**1**) and (**2**).

Compound	(1)	(2)
Empirical formula	C_31_H_34_Cl_2_CuN_14_O_9_	C_33_H_34_Cl_2_CuN_14_O_9_
Formula weight/g/mol	881.16	905.18
Temperature/K	100(2)	173(2)
Crystal system	triclinic	triclinic
Space group	P − 1	P − 1
a/Å	11.6764(3)	11.5914(2)
b/Å	12.1766(3)	12.1725(2)
c/Å	14.4595(3)	15.0547(3)
α/°	99.1420(10)	96.2490(10)
β/°	100.2900(10)	99.9130(10)
γ/°	105.2460(10)	106.1710(10)
Volume/Å^3^	1905.14(8)	1981.87(6)
Z	2	2
ρ_calc_/g/cm^3^	1.536	1.517
μ/mm^−1^	2.721	0.757
F(000)	906	930
Radiation/Å	1.54178	0.71073
Reflections collected	27839	11206
Independent reflections	6708 [R(int) = 0.0367]	7859 [R(int) = 0.0413]
Data/restraints/parameters	6708/0/528	7859/127/591
Goodness-of-fit on F^2^	1.026	1.048
Final R indexes [I ≥ 2σ (I)]	R1 = 0.0330, wR2 = 0.0804	R1 = 0.0529, wR2 = 0.1370
Final R indexes [all data]	R1 = 0.0368, wR2 = 0.0829	R1 = 0.0589, wR2 = 0.1438
Largest diff. Peak/hole/e Å^−3^	0.490/−0.376	0.380/−0.602

**Table 2:** Selected bond distances (Å) and bond angles (º) for (**1**) and (**2**).

(1)							(2)						
Cu1	N1	2.031(2)	N1	Cu1	N2	80.65(6)	Cu1	N1	2.020(2)	N1	Cu1	N2	81.52(9)
Cu1	N2	2.018(2)	N1	Cu1	N31	91.85(7)	Cu1	N2	2.038(2)	N1	Cu1	N31	93.55(9)
Cu1	N21	2.004(2)	N1	Cu1	O1	93.63(6)	Cu1	N21	1.989(2)	N1	Cu1	O1	91.43(9)
Cu1	N31	1.991(2)	N2	Cu1	N21	96.08(7)	Cu1	N31	1.985(2)	N2	Cu1	N21	93.49(9)
Cu1	O1	2.233(2)	N2	Cu1	O1	92.14(6)	Cu1	O1	2.234(2)	N2	Cu1	O1	88.69(9)
			N21	Cu1	N31	90.34(7)				N21	Cu1	N31	90.71(10)
			N21	Cu1	O1	95.48(7)				N21	Cu1	O1	93.47(10)
			N31	Cu1	O1	94.40(6)				N31	Cu1	O1	99.01(10)

**Table 3:** Cyclic voltammetry data for complexes (**1**), (**2**) and [Cu(DMSO)_6_](ClO_4_)_2_.

Complex	E_pa1_(V)	E_pc1_(V)	E_pc2_(V)	E_pc3_(V)	E_1/2_(V) ^a^ (Cu(III)/Cu(II))
**[Cu(DMSO)_6_](ClO_4_)_2_**	+0.431	+0.119			
**(1)**	+0.372	+0.200	+0.009	−0.495	+0.286
**(2)**	+0.412	+0.150	+0.120	−0.700	+0.281

^a^ Half-wave reduction potentials were calculating using E_1/2_ = (E_pa1_ + E_pc1_)/2.

**Table 4:** The half-maximal inhibitory concentration (IC_50_) and therapeutic index (TI) of compounds (**1**), (**2**), and dmtp.

Compound	IC_50_ (µg/mL; μM)				TI	
	BJ		B16			
	24 h	48 h	24 h	48 h	24 h	48 h
**dmtp**	ND *	ND	ND	ND		
**(1)**	ND	ND	5.76; 6.50	3.90; 4.42	8.68	12.82
**(2)**	13.61; 15.04	4.50; 4.97	6.88; 7.60	4.15; 4.58	1.98	1.08

* ND—not determined.

**Table 5:** The IC_50_ (μM) and TI values for perchlorate Cu(II) complexes with N-N and tpds ligands.

Compound	(1)	CubP	CubPP	(2)	CupP	CupPP
**IC_50_ (μM)**	4.42	16	20	4.58	3.70	4.00
**TI**	12.82	5.53	3.23	1.08	0.80	0.88
**Reference**		[29]	[18]		[29]	[18]

CubP, {[Cu(bpy)_2_(μ_2_OClO_3_)]ClO_4_}_n_; CubPP, [Cu(bpy)_2_(pmtp)](ClO_4_)_2_ CupP, [Cu(phen)_2_(OH_2_)](ClO_4_)_2_; CupPP, [Cu(phen)_2_(pmtp)](ClO_4_)_2._

**Table 6:** The MIC values (mM) for ligands and complexes.

Bacterial Strain	dmtp	(1)	(2)
*E. coli* 25922	8.45	0.71	0.34
*P. aeruginosa* 27853	8.45	0.71	0.69
*S. aureus* 25923	8.45	0.18	0.04
MRSA 388	16.90	0.35	0.02

**Table 7:** The compounds’ influence on biofilm formation (MBEC value; mM).

Bacterial Strain	dmtp	(1)	(2)
*E. coli* 25922	8.45	0.71	0.17
*P. aeruginosa* 27853	8.45	0.71	0.69
*S. aureus* 25923	8.45	0.18	0.04
MRSA 388	16.90	0.35	0.04

**Table 8:** The MIC values (mM) for perchlorate Cu(II) complexes with N-N and tpds ligands.

Bacterial Strain	(1)	CubP	CubPP	(2)	CupP	CupPP
*E. coli* 25922	0.71	0.54	0.20	0.34	0.03	0.02
*P. aeruginosa* 27853	0.71	1.09	0.40	0.69	0.50	0.09
*S. aureus* 25923	0.18	0.27	0.40	0.04	0.03	0.01
MRSA 388	0.35	0.27	0.40	0.02	0.12	0.05

**Table 9:** The Cu(II) complexes with N-N and tpds’ influence on biofilm formation (MBEC value; mM).

Bacterial Strain	(1)	CubP	CubPP	(2)	CupP	CupPP
*E. coli* 25922	0.71	0.54	0.40	0.17	0.10	0.02
*P. aeruginosa* 27853	0.71	1.09	0.40	0.69	0.50	0.09
*S. aureus* 25923	0.18	0.14	0.80	0.04	0.03	0.01
MRSA 388	0.35	0.27	0.40	0.04	0.10	0.05

## Data Availability

Not applicable.

## Supplementary Materials

The following are available online, Figure S1: Dimer type formation between two cations trough π⋯π interactions involving the 1,10-phenantroline ligands in (**2**), Figure S2: Dimer-type formation between two dmtp ligands involving CH⋯N interactions in (**2**), Figure S3: Excerpt of the packing diagram of compound (**2**) presenting the formation of linear chains along the *b*-axis trough π⋯π, OH⋯N and CH⋯N interactions between the cations and the dmtp free moieties, Figure S4: IR spectra of dmtp and complexes, Figure S5: UV-Vis spectra of complex (**1**) (dark blue), bpy (yellow) and dmtp (orange), Figure S6: UV-Vis spectra of complex (**2**) (dark blue), phen (yellow) and dmtp (orange), Table S1: Continuous shape measure for the coordination polyhedron around the Cu(II) atom, Table S2: Non-covalent intermolecular interactions in compound **1** (Å and °), Table S3: Non-covalent intermolecular interactions in compound (**2**) (Å and °).

## Sample Availability

Samples of the compounds (**1**) and (**2**) are available from the authors.
