# Supplementary material for: Biological Activity of Triazolopyrimidine Copper(II) Complexes Modulated by an Auxiliary N-N-Chelating Heterocycle Ligands
Source: Molecules. 2021 Nov 9;26(22):6772. doi: 10.3390/molecules26226772 (PMC8620715; doi:10.3390/molecules26226772)
Supplement: Supplementary file 1 [file molecules-26-06772-s001.zip › molecules-1422230-supplementary.pdf]

molecules

MDPI

Supplementary Information

Biological activity of triazolopyrimidine copper(II) complexes modulated by an auxiliary N-N-chelating heterocycle ligands

Lavinia L. Ruţă, Ileana C. Farcaşanu, Mihaela Bacalum, Mina Răileanu, Arpad Mihai Rostas, Constantin-Gabriel Daniliuc, Mariana Carmen Chifiriuc, Luminița Măruțescu, Marcela Popa, Mihaela Badea, Emilia Elena Iorgulescu and Rodica Olar

[1] Department of Organic Chemistry, Faculty of Chemistry, University of Bucharest, Biochemistry and Catalysis, 90–92 Panduri Str., 050663 Bucharest, Romania; lavinia.ruta@chimie.unibuc.ro (L.L.R.); ileana.farcasanu@chimie.unibuc.ro (I.C.F.)

[2] Department of Life and Environmental Physics, Horia Hulubei National Institute for Physics and Nuclear Engineering, 30 Reactorului Str., Măgurele-Ilfov, 077125, Romania; bmihaela@nipne.ro (M.B.); mina.raileanu@nipne.ro (M.R.)

[3] Department of Electricity, Faculty of Physics, University of Bucharest, Solid State and Biophysics, 405A Atomiştilor Str., Măgurele-Ilfov, 077125, Romania

[4] Laboratory of Atomic Structures and Defects in Advanced Materials, National Institute of Materials Physics, 405A Atomiştilor Str., Măgurele-Ilfov, 077125, Romania; arpad.rostas@infim.ro

[5] Organisch-Chemisches Institut, Westfälische Wilhelms-Universität Münster, Corrensstrasse 40, 48149 Münster, Germany; constantin.daniliuc@uni-muenster.de

[6] Department of Microbiology, Faculty of Biology, University of Bucharest, 1–3 Aleea Portocalelor Str., 060101 Bucharest, Romania; carmen.chifiriuc@bio.unibuc.ro (M.C.C.); luminita.marutescu@bio.unibuc.ro (L.M.); marcela.popa@bio.unibuc.ro (M.P.)

[7] Department of Inorganic Chemistry, Faculty of Chemistry, University of Bucharest, Biochemistry and Catalysis, 90–92 Panduri Str., 050663 Bucharest, Romania; mihaela.badea@chimie.unibuc.ro (M.B.); rodica.olar@chimie.unibuc.ro (R.O.)

[8] Department of Analytical Chemistry, Faculty of Chemistry, University of Bucharest, 90–92 Panduri Str., 050663 Bucharest, Romania; emilia-elena.iorgulescu@chimie.unibuc.ro

* Correspondence: ileana.farcasanu@chimie.unibuc.ro (I.C.F); rodica.olar@chimie.unibuc.ro (R.O.)

X-ray crystal structure analysis of (1): A blue prism-like specimen of $C_{31}H_{34}Cl_2CuN_{14}O_9$, with approximate dimensions of 0.140 mm x 0.280 mm x 0.320 mm, was used for the X-ray crystallographic analysis. The X-ray intensity data were measured. A total of 1309 frames were collected. The total exposure time was 8.42 hours. The frames were integrated with the Bruker SAINT software package using a wide-frame algorithm. The integration of the data using a triclinic unit cell yielded a total of 27839 reflections to a maximum θ angle of 66.71° (0.84 Å resolution), of which 6708 were independent (average redundancy 4.150, completeness = 99.5%, R_{int} = 3.67%, R_{sig} = 3.08%) and 6109 (91.07%) were greater than 2σ(F^2). The final cell constants of a = 11.6764(3) Å, b = 12.1766(3) Å, c = 14.4595(3) Å, α = 99.1420(10)°, β = 100.2900(10)°, γ = 105.2460(10)°, and volume = 1905.14(8) $Å^3$, were based upon the refinement of the XYZ-centroids of 9913 reflections above 20 σ(I) with 6.364° < 2θ < 133.3°. Data were corrected for absorption effects using the multi-scan method (SADABS). The ratio of minimum to maximum apparent transmission was 0.792. The calculated minimum and maximum transmission coefficients (based on crystal size) were 0.4760 and 0.7020. The structure was resolved and refined using the Bruker SHELXTL Software Package, using the space group *P*-1, with Z = 2 for the formula unit, $C_{31}H_{34}Cl_2CuN_{14}O_9$. The final anisotropic full-matrix least-squares refinement of F^2 with 528 variables converged at R1 = 3.30% for the observed data, and wR2 = 8.29% for all the data. The goodness-of-fit was 1.026. The largest peak in the final difference electron density synthesis was 0.490 $e^-/Å^3$ and the largest hole was -0.376 $e^-/Å^3$, with an RMS deviation of 0.059 $e^-/Å^3$. On the basis of the final model, the calculated density was 1.536 g/cm³ and F(000), 906 e^-. The hydrogen atoms at O1 were refined freely. CCDC number: 2067846.

X-ray crystal structure analysis of (2): A blue plate-like specimen of $C_{33}H_{34}Cl_2CuN_{14}O_9$, with approximate dimensions of 0.050 mm x 0.120 mm x 0.180 mm, was used for the X-ray crystallographic analysis. The X-ray intensity data were measured. The integration of the data using a triclinic unit cell yielded a total of 11206 reflections to a maximum θ angle of 26.37° (0.80 Å resolution), of which 7859 were independent (average redundancy 1.426, completeness = 96.8%, R_{int} = 4.13%, R_{sig} = 4.25%) and 7078 (90.06%) were greater than 2σ(F^2). The final cell constants of a = 11.5914(2) Å, b = 12.1725(2) Å, c = 15.0547(3) Å, α = 96.2490(10)°, β = 99.9130(10)°, γ = 106.1710(10)°, and volume = 1981.87(6) $Å^3$, were based upon the refinement of the XYZ-centroids of reflections above 20 σ(I). Data were corrected for absorption effects using the multi-scan method (SADABS). The calculated minimum and maximum transmission coefficients (based on crystal size) were 0.8760 and 0.9630. The structure was resolved and refined using the Bruker SHELXTL Software Package, using the space group *P*-1, with Z = 2 for the formula unit, $C_{33}H_{34}Cl_2CuN_{14}O_9$. The final anisotropic full-matrix least-squares refinement of F^2 with 591 variables converged at R1 = 5.29% for the observed data, and wR2 = 14.38% for all the data. The goodness-of-fit was 1.048. The largest peak in the final difference electron density synthesis was 0.380 $e^-/Å^3$ and the largest hole was -0.602 $e^-/Å^3$, with an RMS deviation of 0.082 $e^-/Å^3$. On the basis of the final model, the calculated density was 1.517 g/cm^3 and F(000), 930 e^-. The hydrogen atoms at O1 were refined freely. CCDC number: 2067847.

Table S1. Continuous shape measurements for the coordination polyhedron around the Cu(II) atom.

Geometry	(1)	(2)
PP-5	31.295	30.613
vOC-5	0.670	0.604
TBPY-5	5.854	5.947
SPY-5	0.795	0.897
JTBPY-5	8.317	8.279

Table S2. Non-covalent intermolecular interactions in compound **1** (Å and °).

D*-H···*A	***d*(*D*-H)**	***d*(H···*A*)**	***d*(*D*···*A*)**	**∠(*DHA*)**
O1-H1A···N11	0.78(4)	2.20(4)	2.947(2)	161(4)
C9-H9···O22	0.95	2.62	3.110	112.7
C10-H10···N21	0.95	2.63	3.162	115.6
C21-H21···O14	0.95	2.43	3.281	149.6
C1-H1···O21$^{\#1}$	0.95	2.50	3.260	136.7
C2-H2···O22$^{\#1}$	0.95	2.59	3.430	148.2
C7-H7···N34$^{\#2}$	0.95	2.66	3.483	144.6
C11-H11···N12$^{\#3}$	0.95	2.52	3.286	137.4
C17-H17c···O23$^{\#1}$	0.98	2.46	3.437	173.4
C24-H24···O12$^{\#4}$	0.95	2.55	3.424	152.9
C26-H26c···O13$^{\#5}$	0.98	2.61	3.498	150.0
C34-H34···O11$^{\#6}$	0.95	2.49	3.427	167.6
*Cg*1···*Cg*1$^{\#3}$				3.377
*Cg*2···*Cg*3				3.465

Symmetry transformations used to generate equivalent atoms: $^{\#1}$ x−1, y, z; $^{\#2}$ -x + 1, −y + 1, −z + 2; $^{\#3}$ -x + 1, −y + 2, −z + 2; $^{\#4}$ x, y−1, z; $^{\#5}$ -x + 1, −y + 1, −z + 1; $^{\#6}$ x−1, y−1, z. *Cg*1 is the centroid involving the entire 2,2′-bipyridine ligand. *Cg*2 is the centroid involving the atoms N1/C1/C2/C3/C4/C5 of the 2,2′-bipyridine ligand. *Cg*3 is the centroid involving the atoms N11/C11/N12/C12/N13 of the 5,7-dimethyl-1,2,4-triazolo[1,5-*a*]pyrimidine moieties.

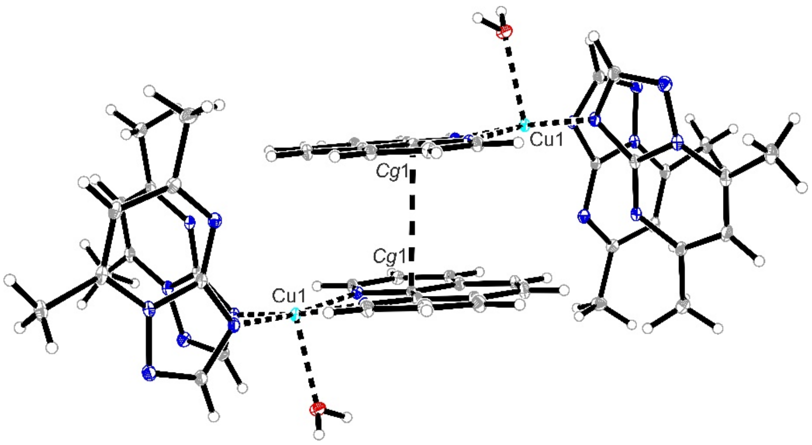

Figure S1. Dimer type formation between two cations trough π···π interactions involving the 1,10-phenantroline ligands in (**2**).

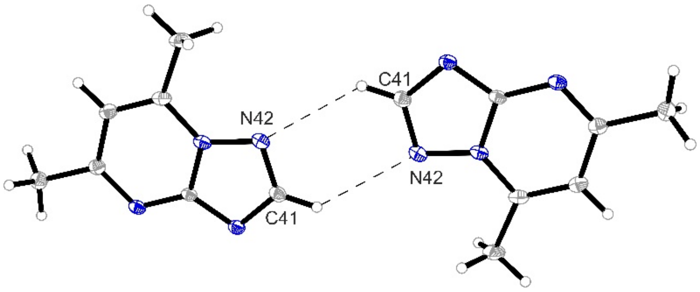

Figure S2. Dimer type formation between two dmtp ligands involving CH···N interactions in (**2**).

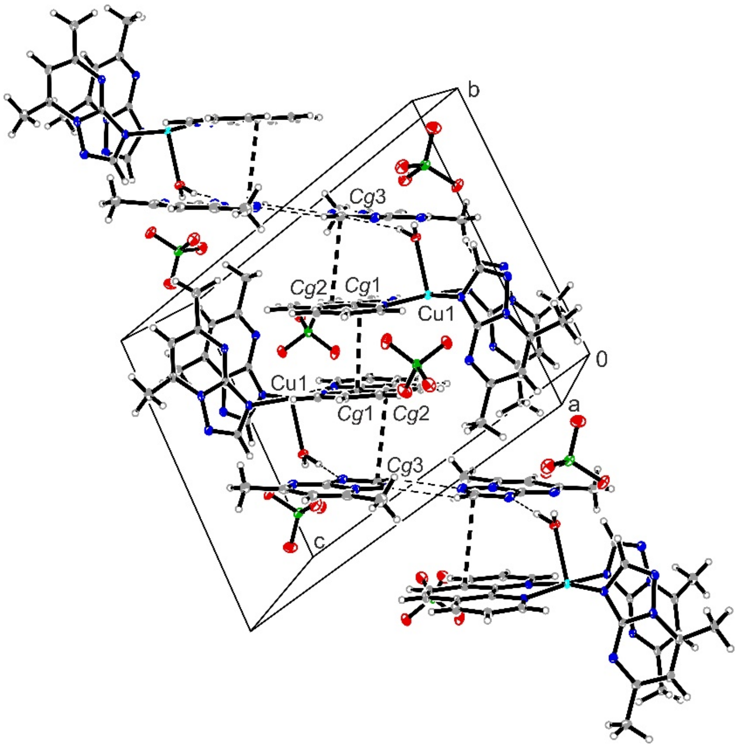

Figure S3. Excerpt of the packing diagram of compound (**2**) presenting the formation of linear chains along the *b*-axis trough π···π, OH···N and CH···N interactions between the cations and the dmtp-free moieties.

Table S3. Non-covalent intermolecular interactions in compound (**2**) (Å and °).

D*-H···*A	***d*(*D*-H)**	***d*(H···*A*)**	***d*(*D*···*A*)**	**∠(*D*H*A*)**
O1-H1A···O12 $^{\#1}$	0.81(4)	166(4)	2.04(4)	2.833(4)
O1-H1B···N41$^{\#1}$	0.93(4)	154(4)	2.02(4)	2.700(2)
C2-H2···O21	0.95	135.2	2.48	3.118(4)
C1-H1···N31	0.95	112.9	2.64	3.130(4)
C11-H11···O21$^{\#2}$	0.95	122.4	2.50	3.118(4)
C12-H12···O21$^{\#2}$	0.95	123.0	2.51	3.131(4)
C21-H21···N44$^{\#1}$	0.95	125.5	2.69	3.329(4)
C24-H24···O14$^{\#2}$	0.95	156.9	2.38	3.278(6)
C31-H31···O13$^{\#1}$	0.95	137.6	2.56	3.325(6)
C34-H34···O11	0.95	156.9	2.49	3.386(4)
C37-H37A···O12$^{\#3}$	0.98	161.0	2.59	3.530(5)
C41-H41···N42$^{\#4}$	0.95	133.2	2.66	3.385(5)
C46-H46B···O14A$^{\#2}$	0.98	148.2	2.39	3.260(3)
C46-H46C···O22$^{\#5}$	0.98	161.2	2.65	3.591(5)
*Cg*1···*Cg*1$^{\#6}$				3.594
*Cg*2···*Cg*3$^{\#4}$				3.463

Symmetry transformations used to generate equivalent atoms: $^{\#1}$ x, y + 1, z; $^{\#2}$ x−1, y, z; $^{\#3}$ −x + 1, −y + 1, −z; $^{\#4}$ −x + 1, −y + 1, −z + 1; $^{\#5}$ x−1, y−1, z; $^{\#6}$ −x + 1, −y + 2, −z + 1. *Cg*1 is the centroid involving the entire 1,10-phenantroline ligand. *Cg*2 is the centroid involving the atoms N2/C8/C9/C10/C11/C12 of the 1,10-phenantroline ligand. *Cg*3 is the centroid involving the atoms N41/C41/N42/C42/N43 of the 5,7-dimethyl-1,2,4-triazolo[1,5-*a*]pyrimidine moieties.

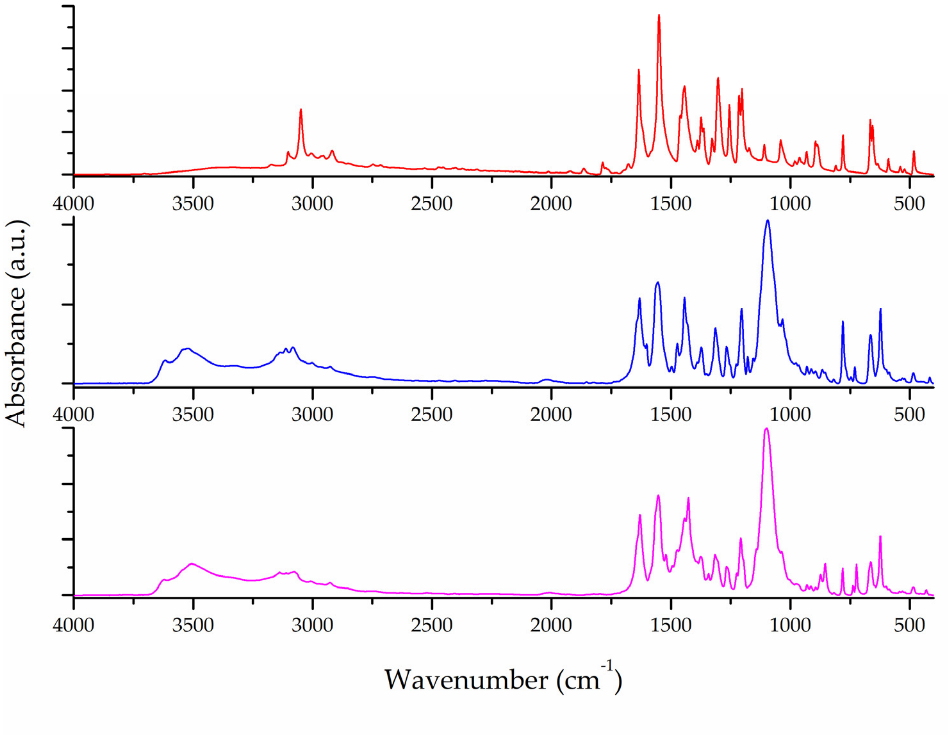

Figure S4. IR spectra of dmtp and complexes.

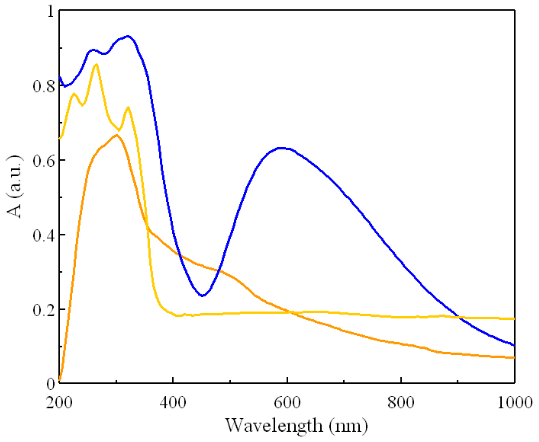

Figure S5. UV-Vis spectra of complex (**1**) (dark blue), bpy (yellow) and dmtp (orange).

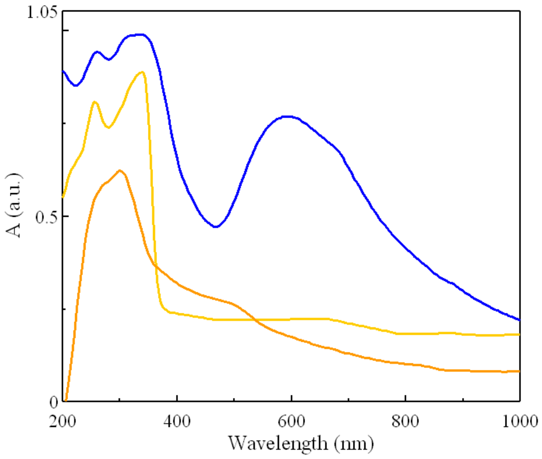

Figure S6. UV-Vis spectra of complex (**2**) (dark blue), phen (yellow) and dmtp (orange).

References

1. APEX3 (**2016**), SAINT (**2015**) and SADABS (**2015**), Bruker AXS Inc., Madison, Wisconsin, USA.
2. Sheldrick, G. M. SHELXT – Integrated space-group and crystal-structure determination. *Acta Crystallogr*. **2015**, *A71*, 3-8, https://doi.org/10.1107/S2053273314026370.
3. Sheldrick, G.M. Crystal structure refinement with SHELXL. *Acta Crystallogr*. **2015**, *C71*, 3-8, https://doi.org/10.1107/S2053229614024218.
4. Hooft, R. W. W.; Nonius B. V. *COLLECT, Program for Collecting Data on CCD Area Detectors*, **1998**, Delft, The Netherlands.
5. Otwinowski, Z.; Minor, W. Processing of X-ray diffraction data collected in oscillation mode. *Methods Enzymol*. **1997**, *276*, 307-326, https://doi.org/10.1016/S0076-6879(97)76066-X.
6. Otwinowski, Z.; Borek, D.; Majewski, W.; Minor, W. Multiparametric scaling of diffraction intensities. *Acta Crystallogr*. **2003**, *A59*, 228-234, https://doi.org/10.1107/S0108767303005488.
7. XP – Interactive molecular graphics, Version 5.1, Bruker AXS Inc., Madison, Wisconsin, USA, **1998**.
